# Aquaculture Breeding Enhancement: Maturation and Spawning in Sea Cucumbers Using a Recombinant Relaxin-Like Gonad-Stimulating Peptide

**DOI:** 10.3389/fgene.2019.00077

**Published:** 2019-02-19

**Authors:** Hoang Dinh Chieu, Luke Turner, Meaghan K. Smith, Tianfang Wang, Josephine Nocillado, Peter Palma, Saowaros Suwansa-ard, Abigail Elizur, Scott F. Cummins

**Affiliations:** ^1^Genecology Research Centre, University of the Sunshine Coast, Sippy Downs, QLD, Australia; ^2^Research Institute for Marine Fisheries, HaiPhong, Vietnam; ^3^Tasmanian Seafoods Pty. Ltd., Smithton, TAS, Australia; ^4^Aquaculture Department, Southeast Asian Fisheries Development Center, Iloilo, Philippines

**Keywords:** aquaculture, juvenile, oocyte maturation, recombinant RGP, sea cucumbers, spawning

## Abstract

Wild sea cucumber resources have been rapidly exhausted and therefore there is an urgent need to develop approaches that will help restocking. Currently, there is a lack of information regarding the genes involved in sea cucumber reproductive processes. The neurohormone relaxin-like gonad-stimulating peptide (RGP) has been identified as the active gonad-stimulating peptide in sea stars (Asteroidea), which could also be present in other echinoderm groups. In this study, a sea cucumber RGP was identified and confirmed by phylogenetic analysis. A recombinant *Holothuria scabra* RGP was produced in the yeast *Pichia pastoris* and confirmed by mass spectrometry. To assess bioactivity, four levels of purification were tested in an *in vitro* germinal vesicle breakdown (GVBD) bioassay. The most pure form induced 98.56 ± 1.19% GVBD in *H. scabra* and 89.57 ± 1.19% GVBD in *Holothuria leucospilota*. Cruder levels of purification still resulted in some GVBD. Upon single injection into female *H. scabra*, the recombinant RGP induced head waving behavior followed by spawning within 90–170 min. Spawned oocytes were fertilized successfully, larvae settled and developed into juveniles. Our results provide a key finding for the development of a break-through new artificial breeding approach in sea cucumber aquaculture.

## Introduction

Sea cucumbers are widely considered as commercially valuable with a high demand for consumption and use in some traditional medicines (Olivera-Castillo et al., 2013; Fahmy et al., 2015). As a result, many species have been critically overfished, which has led to the rapid exhaustion of wild populations (Lovatelli, 2004; Purcell et al., 2011). With reduced stocks, artificial breeding has become an effective solution to produce high quality seed for aquaculture, stock enhancement, and sea ranching. Spawning induction is a critical procedure in artificial breeding, however, traditional stimulation methods such as thermal shock, drying then rehydration, exposure to conspecific sperm, and a combination of the aforementioned treatments (Al Rashdi et al., 2012) are not efficient. In addition, traditional stimulation methods are thought to reduce the health of broodstock. As such, the employment of reproductive neuropeptide hormones for achieving reproductive maturation and spawning is an innovative approach with great potential to advance hatchery production in sea cucumber aquaculture.

In sea cucumbers, immature oocytes cannot be fertilized and insemination is only successful following germinal vesicle breakdown (GVBD), leading to the formation of the first and second polar bodies (Maruyama, 1980). Oocyte maturation and ovulation can be induced by a hormonal substance present within the radial nerve (Maruyama, 1980). Crude radial nerve extract (RNE) has been shown to stimulate maturation of fully grown oocytes within various sea cucumber species, including *Holothuria leucospilota, Holothuria pervicax, Holothuria moebi, Holothuria pardalis*, and *Apostichopus japonicus* (Maruyama, 1980, 1985; Katow et al., 2009). Meanwhile, several peptide hormones have been recognized as inducers of oocyte maturation in sea cucumbers, including the gonad-stimulating substance-like molecule (GSSL), cubifrin (NGIWYamide, NGLWYamide), and the heptapeptide QGLFSGVamide (Kato et al., 2009; Katow et al., 2009; Leonet et al., 2009; Fujiwara et al., 2010; Yamano et al., 2013).

GSSL is a 4.8 kDa single chain peptide which was isolated from the radial nerve of *A. japonicus*. A synthetic GSSL peptide, at 6 μM, was capable of inducing GVBD at 50% efficiency (Katow et al., 2009). The neuropeptide cubifrin-I (NGIWYamide) and its derivative cubifrin-L (NGLWYamide) have been reported as potent inducers of *in vitro* oocyte maturation and spawning in *A. japonicus* (Kato et al., 2009; Fujiwara et al., 2010; Yamano et al., 2013). However, cubifrin may not be effective for all sea cucumber species, where for example, synthetic cubifrin could not induce oocyte maturation in *H. leucospilota* at concentrations from 1 nM to 10 μM (Chieu et al., 2018). Meanwhile, a second synthesized peptide, QGLFSGVamide could induce GVBD at concentrations ≥1 μM (Kato et al., 2009). However, due to its low potency, this peptide was not suggested as the primary endocrine regulator of oocyte maturation and therefore was not considered for further study (Kato et al., 2009). Lastly, MIF (maturation inducing fractions) collected by gel filtration chromatography from spawned egg extract of the regular sea urchins (e.g., *Echinometra mathaei, Stomopneustes variolaris, Paracentrotus lividus*) could also induce oocyte maturation and spawning in the sea cucumber (Leonet et al., 2009). The bioactive peptide sequences of MIF are still unknown.

The relaxin-like gonad-stimulating peptide (RGP), originally denoted as GSS (gonad-stimulating substance) in the sea stars due to its classification as a member of the relaxin superfamily, is suggested as the first identified invertebrate gonadotropin to trigger final gamete maturation (Mita et al., 2009; Mita, 2013, 2016). The neuroendocrine mechanism involved in endogenous regulation of oocyte maturation and spawning is considered to be similar among other echinoderm groups, including the sea cucumbers (Smiley, 1990; Mercier et al., 2009). RGP is a heterodimer composed of two peptides (A- and B-chains) with disulphide cross-linkages and the A-chain harboring a cysteine motif “CCXXXCXXXXXXXXC”, where “X” represents any other amino acid (Mita et al., 2009; Mita, 2013). Synthetic RGP (bonded) has been shown to induce oocyte maturation and ovulation within 30 min of incubation, however, no induction occurred when ovarian fragments were incubated with A- or B-chains alone, or as a mixture (unbonded) (Mita et al., 2009; Mita, 2013). As a result, the quaternary structure of RGP is necessary for hormonal action and this structure is complex and difficult to chemically synthesize. Instead of synthetic RGP, an effective approach to obtain RGP with correct quaternary structure is the production of a recombinant RGP in the methylotrophic yeast such as *Hansenula polymorpha, Pichia pastoris*, and *Candida boidinii*.

The methylotrophic yeast (*P. pastoris*) has been developed as a high-level production system for recombinant proteins (Hollenberg and Gellissen, 1997), with several advantages such as rapid growth rate, high levels of productivity in an almost protein-free medium, ease of genetic manipulation of well-characterized yeast expression vectors and diverse post-translational modifications of disulphide bonds within the yeasts secretory pathway (Weinacker et al., 2013). Recombinant gonadotropin or gonad-stimulating hormone like peptides have been produced and studied in several aquatic species, such as a recombinant tilapia LH stimulated the release of 11-ketotestosterone from mature testes (Kasuto and Levavi-Sivan, 2005); a recombinant molt-inhibiting hormone-B (MeMIH-B) that could induce yolk protein synthesis in the shrimp (*Metapenaeus ensis*) (Tiu and Chan, 2007); induction of spermatogenesis in the eel *Anguilla japonica* by a recombinant goldfish (*Carassius auratus*) gonadotropins (Hayakawa et al., 2008); regulation of steroidogenesis and early ovarian development in juvenile grouper by a recombinant orange-spotted grouper (*Epinephelus coioides*) follicle-stimulating hormone (FSH) (Chen et al., 2012); phosphorylation was elevated significantly in the testis of Eastern spiny lobster *Sagmariasus verreauxi* by a recombinant insulin-like androgenic gland hormone (Aizen et al., 2016); and *in vitro* and *in vivo* biological activities in oocyte development and spermatogenesis by a recombinant yellowtail kingfish (*Seriola lalandi*) FSH (Sanchis-Benlloch et al., 2017). However, production and biological activities of recombinant gonadotropin or gonad-stimulating hormones/peptides have been poorly studied in invertebrates, especially in echinoderm species.

RGP has not been well studied in the sea cucumbers with the exception of the first identification of relaxin-like peptide-1 (gonad-stimulating substance type) genes reported in *Holothuria scabra, Holothuria glaberrima* and *A. japonicus* (Suwansa-ard et al., 2018). To date, the bioactive functions of RGP have not been verified in sea cucumbers. In this study, we have investigated the phylogeny of sea cucumber RGPs with other echinoderm species relaxin-like peptides. A recombinant *H. scabra* RGP was generated in the *P. pastoris* yeast expression system and subsequently confirmed by mass spectrometry. The recombinant RGP, when injected, induced oocyte maturation and spawning and the spawned oocytes were successfully fertilized leading to larvae and juvenile development.

## Materials and Methods

### RGP Annotation and Comparative Sequence Analysis

Transcripts encoding echinoderm RGP were obtained by tBLASTn search using the *H. scabra* RGP (Suwansa-ard et al., 2018) as a query. The deduced amino acid sequence of *H. leucospilota* RGP was analyzed for the presence of a signal peptide by using the SignalP 4.1 (Petersen et al., 2011). Proteomic cleavage sites and post-translational modifications of the RGP precursor peptide were predicted by NeuroPred (Southey et al., 2006). Amino acid alignment of echinoderm RGP was performed by MEGA 7 software (Kumar et al., 2016) and subsequently illustrated by using MikTex Texshade software. Phylogenetic tree analysis of echinoderm RGP based on maximum likelihood estimation was conducted using the Phylogeny.fr webserver (http://www.phylogeny.fr). The tree was constructed by using the amino acid sequences of mature RGP A- and B-chain peptides with the inter-chain region, along with its evolutionary-related peptide, the insulin-like growth factor (IGF), which was used as an outgroup. The parameters were set as follows: substitution model, WAG; gamma, 2.5; Number of substitution rate categories, 4; number of bootstraps, 500.

### Construction and Transformation of RGP Recombinant Plasmid

**Figure 1:**
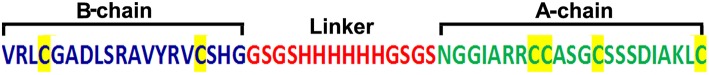
*Holothuria scabra* RGP protein encoded within the vector construct. A-Chain (green), B-chain (Blue) and histidine linker (red) regions are shown. Yellow highlight backgrounds indicate conserved cysteine residues.

A *H. scabra* RGP construct was designed with a histidine linker positioned between chain A and chain B (Figure 1). Following synthesis and insertion of the RGP gene into pPIC9K vector (Genscript Biotech Company), it was transformed into JM109 competent cells (Promega Corporation). Recombinant plasmid RGP-pPIC9K was then purified using the GeneJET plasmid Midiprep Kit (Thermo Scientific) and digested with Sal I (Thermo Scientific) using a standard approach, before being transformed into the yeast *P. pastoris* (strain SuperMan5, phenotype His^−^, Biogrammatics) following a standard electroporation protocol (Madden et al., 2015). Transformed yeast cells were grown on RDB (regeneration dextrose medium) plates for 4 days at 30°C. After that, colonies were transferred to 96-well culture plates containing 200 μl yeast extract:peptone:dextrose (YPD) solution/well supplemented with different concentrations of Geneticin selective antibiotic (G418 disulfate, MERCK), including at 0, 1, and 2 mg/mL, respectively. After growing at 30°C for 1–2 days, RGP colony growth was determined at OD_600_ using a Multimode plate Reader (EnSpire 2300, Perkin Elmer).

### Induction of Recombinant Protein Expression

Yeast containing recombinant RGP plasmid with high expression levels were inoculated into a 200 mL YPD solution, at 28°C. After 24 h, yeast cells were collected by centrifugation at 3,000 x g, for 5 min and subsequently resuspended in 400 mL BMG (buffered minimal glycerol) medium and grown for 24 h, at 28°C, until an OD_600_ >1. Then, cells were pelleted by centrifugation (3,000 × g, for 5 min at 28°C) and resuspended in BMM (buffered minimal methanol) medium. After 3 days, cells were collected by centrifugation (3,000 × g, for 10 min at 4°C). The supernatant was taken for secreted protein analysis. For negative controls, yeast cells containing no recombinant plasmid were grown as described and the supernatant collected for secreted protein analysis.

### Recombinant RGP Purification

Recombinant RGP was purified to 4 levels:

1. Crude RGP supernatant (CS): supernatant was collected by centrifugation (3,000 x g, for 10 min at 4 °C) after growing yeast in BMM solution for 3 days.
2. Crude concentrated RGP (CR): CS was immediately frozen in liquid nitrogen, and then lyophilized by freeze-drying (model ModulyoD, Thermo Fisher).
3. C18 SepPak-purified RGP (SR): CS was purified through C18 SepPak cartridge (Waters Corporation) following the manufacturer's instruction.
4. His and Amicon-purified RGP (HR): CS was treated using a Ni-NTA superflow (QIArack kit, QIAGEN) under native conditions. Imidazole was removed from elution fractions using an Amicon Ultra-15 centrifugal filter device with a 3 KDa cut-off (MERCK). All purification steps followed the manufacturers guidelines.

Protein concentration was determined using a Nanodrop 2000 (Thermo Scientific, Wilmington, DE, USA). All preparations were kept at−80°C before used in mass spectrometry analysis and bioassays.

### uHPLC Tandem QTOF MS/MS Analyses of Recombinant RGP

His and Amicon-purified RGP was digested by trypsin (Promega) in-solution using the method described previously (Ni et al., 2018). Then, trypsin-digested peptide solutions were adjusted to pH <3 by adding 10% formic acid and analyzed by LC-MS/MS on an ExionLC liquid chromatography system (AB SCIEX) coupled to a QTOF X500R mass spectrometer (AB SCIEX) equipped with an electrospray ion source. Twenty microliters of each sample were injected onto an Aeris™ 1.7 μm PEPTIDE XB-C18 100 Å uHPLC column (Phenomenex, Sydney, Australia) equipped with a SecurityGuard column for mass spectrometry analysis. For mobile phases, solvent A consisted of 0.1% (v/v) formic acid and solvent B contained 100% acetonitrile/0.1% formic acid. Linear gradients of 5–35% solvent B over 10 min at 400 μL/min flow rate, followed by a steeper gradient from 35 to 80% solvent B in 2 min and 80 to 95% solvent B in 1 min were used for peptide elution. Solvent B was held at 95% for 1 min for washing the column and returned to 5% solvent B for equilibration prior to the next sample injection. The ionspray voltage was set to 5500V, declustering potential 100V, curtain gas flow 30, ion source gas 1 40, ion source gas 2 (GS2) 50 and spray temperature at 450°C. The mass spectrometer acquired mass spectral data in an Information Dependant Acquisition, IDA mode. Full scan TOF-MS data was acquired over the mass range 350–1,400 and for product ion ms/ms 50–1,800. Ions observed in the TOF-MS scan exceeding a threshold of 100 cps and a charge state of +2 to +5 were set to trigger the acquisition of product ion. The data was acquired and processed using SCIEX OS software (AB SCIEX, Concord, Canada). Biological triplicates were used for the analysis.

LC-MS/MS data was imported into the PEAKS studio (Bioinformatics Solutions Inc., version 7.0) with the assistance of MS Data Converter (Beta 1.3, http://sciex.com/software-downloads-x2110). *De novo* sequencing of peptides, database search and characterizing specific post translational modifications (PTMs) were used to analyse the raw data; false discovery rate was set to ≤ 1%, and [-10^*^log(p)] was calculated accordingly where p is the probability that an observed match is a random event. The PEAKS used the following parameters: (i) precursor ion mass tolerance, 0.1 Da; (ii) fragment ion mass tolerance, 0.1 Da; (iii) tryptic enzyme specificity with two missed cleavages allowed; (iv) monoisotopic precursor mass and fragment ion mass; (v) a fixed modification of cysteine carbamidomethylation; and (vi) variable modifications including lysine acetylation, deamidation on asparagine and glutamine, oxidation of methionine and conversion of glutamic acid and glutamine to pyroglutamate.

### Structure Prediction of Recombinant RGP Using Molecular Dynamic Simulation

The initial conformations of recombinant RGP were built as a linear structure using the LEAP module of AMBER 14 (Case et al., 2014). Molecular dynamic (MD) simulation was fully unrestrained and carried out in the canonical ensemble using the SANDER module. The ff14SB force field (Duan et al., 2003) was employed. Energy minimization with 2500 steps was first performed to remove unfavorable contacts. The AMBER structure was then heated to 325K over 50 ps to avoid being kinetically trapped in local minima, then subjected to unrestrained MD simulations at 325K for the purpose of peptide equilibration. The structural information was sampled every 1 ps (i.e., 10,000 structures were calculated for 10 ns MD simulation). This MD simulation was continued until the root mean square deviation of structures within a reasonable long time range was stable at/less than 3~4Å. Then a lowest energy structure was determined and considered as the representative of the conformations simulated over this period. Visualization of the systems was effected using VMD software (Humphrey et al., 1996).

### Germinal Vesicle Breakdown and Spawning Bioassay Using Recombinant RGP

Adult female *H. scabra* (300 ± 50 g in body weight) were obtained from a broodstock population at a commercial aquaculture facility within the Darwin Aquaculture Center (Northern Territory, Australia). Adult female *H. leucospilota* (250 ± 50 g in body weight) based on biopsy via a small incision at the dorsal bivium, were collected from Point Cartwright (Mooloolaba, Australia) and housed in an protein skimmed saltwater aquarium system at the University of the Sunshine Coast (Sippy Downs, Australia).

For the *in vitro* GVBD bioassay, ovarian tubules were dissected from individuals of *H. scabra* and *H. leucospilota* at gonad stage III (advanced mature, oocyte diameter > 150 μm). Ovarian tubules were cut transversely into ~5 mm long sections and transferred into 96-well microplates containing 80 μL of filtered artificial sea water (FASW) and different RGP extracts, including CS, CR, SR and HR, at various concentrations as follows: 1.75–7.00 μg/μL of CS; 28.25–113.00 μg/μL of CR; 24.00–96.00 μg/μL of SR; and 0.004–0.016 μg/μL of HR. Yeast supernatant containing no recombinant plasmid was used as a negative control, while 0.2 μg/μL RNE prepared as described in our previous study (Chieu et al., 2018) was used as a positive control. Each test sample was performed in triplicate. Ovarian tubules were incubated in the test media at 24°C for 3 h and then released oocytes and in-tubule oocytes identified under a stereomicroscope (Nikon SMZ800N, Nikon). Observations were taken regularly during this period. The total number of mature and immature oocytes was recorded based on presence or absence of the germinal vesicle and recorded as a percentage.

For the *in vivo* spawning bioassay, CS and HR were tested for *H. scabra* behavior changes and induction of spawning. Two negative controls, including filtered artificial seawater (FASW) and yeast supernatant without RGP (YR), were also included. Twelve sea cucumbers (ca. 250 g in total body weight/animal), 3 for each test sample, were kept separately in 5 L seawater. Test samples were injected into the body wall. The injection schedule and concentrations for each test sample is shown in Table 1. Animal behavior, specifically head waving (where the anterior region sways from side to side at the surface of the seawater) that is a typical behavior for sea cucumbers prior to spawning and spawning were recorded using time-lapse (HD webcam 720p, Logitech) for 1.5 h and if no spawning was observed, a second injection was performed.

**Table 1:** Schedule and concentration for the tested sample injections into female *Holothuria scabra*.

**Test sample**	**1st injection (2 mL) (mg peptide/animal)**	**2nd injection (3 mL) (mg peptide/animal)**
Crude RGP supernatant (CS)	14	21
His and Amicon-purified RGP (HR)	0.040	NA
Filtered artificial seawater (FASW)	0	0
Yeast supernatant without RGP (YR)	0	0

Spawned oocytes were collected for fertilization with spermatozoa aspirated from a mature *H. scabra* using a hypodermic needle and syringe. The spermatozoa were suspended in 50 mL seawater, from there, a few drops of sperm solution were added to containers with mature oocytes. After 30 min, fertilized ova were collected by filtering using a 60 μm mesh screen and the excess spermatozoa removed by rinsing with seawater. Fertilized ova were hatched in 1,000 L tanks and transferred into 5,000 L rearing tanks at 2 days post-fertilization. The larvae were fed daily with live diatoms until reaching juvenile stage.

## Results

### Comparative Analysis of Echinoderm RGPs and IGFs

Echinoderm RGP transcripts present within the NCBI databases were obtained, including a single transcript encoding RGP peptide precursor deduced from *H. leucospilota* (Supplementary Data S1). The *H. leucospilota* RGP peptide precursor is composed of 123 amino acid residues, which includes a 25 amino acid signal peptide sequence and a 98 amino acid RGP propeptide that contains two predicted A- and B-chain RGP peptides. The A-chain peptide, located at the C-terminus of the RGP propeptide, is 22 residues in length, while the B-chain peptide, located at the N-terminus of the RGP propeptide, is 19 residues in length and includes a C-terminal glycine residue (predicted to be an amide donor for C-terminal amidation). Both A- and B-chain peptides contain highly conserved cysteine residues that are known to be essential for disulphide bridge formation.

**Figure 2:**
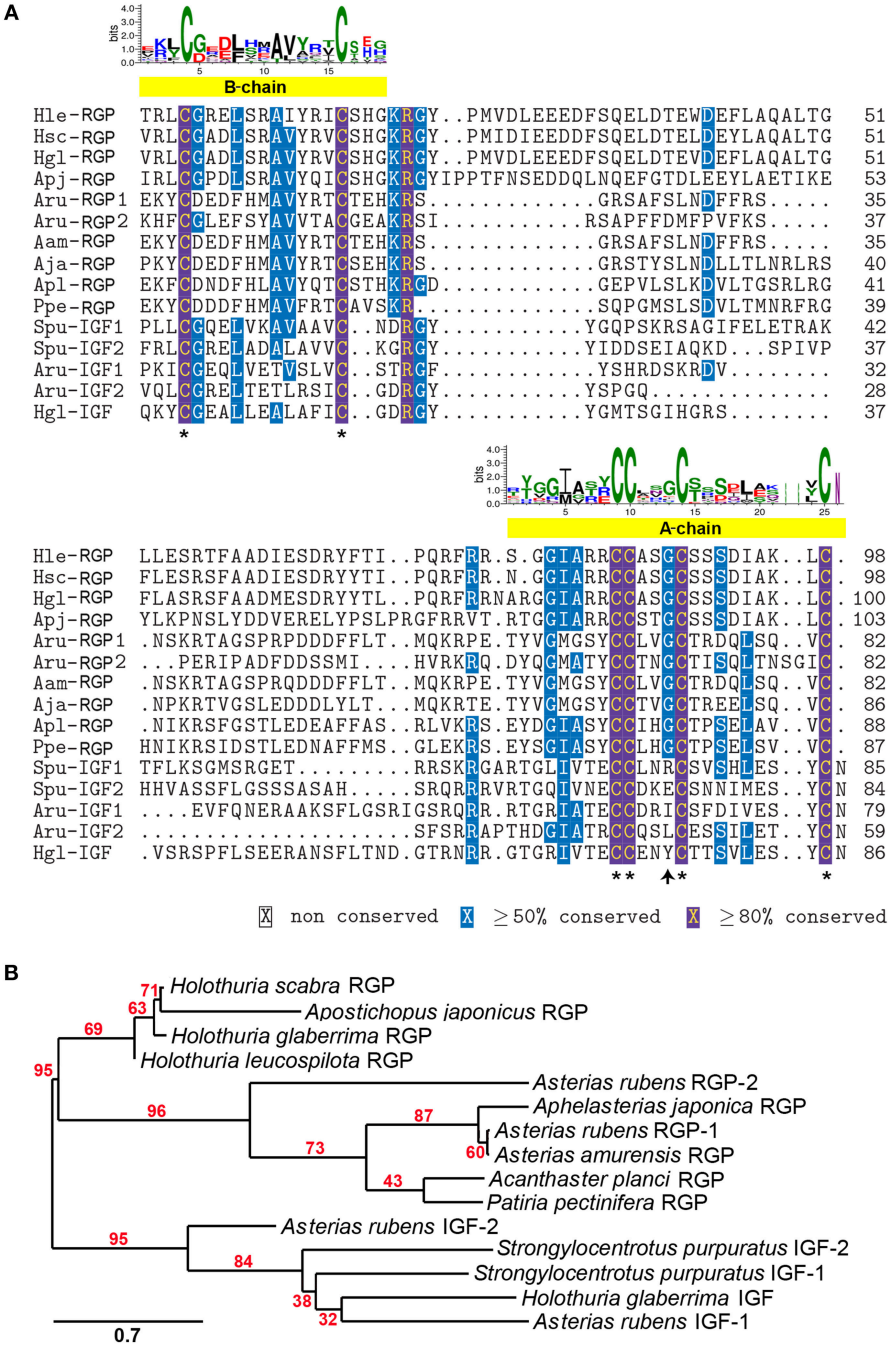
Multiple sequence alignment and phylogenetic tree analysis of echinoderm RGP/IGF mature peptides. **(A)** Alignment of echinoderm RGP/IGF mature peptides. The yellow bars indicate the putative A- and B-chain peptides which contain the highly conserved cysteine residues (asterisks) important for disulphide bridge formation. The sequence logo above the alignment shows the conservation of amino acid composition at each position. **(B)** Phylogenetic tree analysis of sea cucumber and other echinoderm RGP mature peptides based on maximum likelihood estimation (500 bootstraps). The echinoderm IGFs were used as the outgroup. The numbers above nodes indicate the branch support values. The scale bar indicates the estimated amino acid substitutions per site. For species abbreviations and sequences used in the amino acid alignment and phylogenetic tree, see Supplementary Data S1.

A comparative amino acid alignment of echinoderm RGPs/IGFs was performed (Figure 2A). *H. leucospilota* RGP shows the highest similarity in its amino acid composition to the *H. glaberrima* RGP (87.8% identity) and, with a lesser extent, the *H. scabra* RGP (86.99%). However, *H. leucospilota* RGP shows only 49.59% identity to the sea cucumber *A. japonicus*. Although RGPs and IGFs share the conservation of amino acid residues within the A- and B-chain regions, especially the conserved cysteine residues which are potentially important for disulphide bridge formation (asterisks, Figure 2A), variable amino acids were observed throughout the mature peptide length. Noteworthy, the glycine residues located prior to the third conserved cysteine residue of the A-chains were present in RGPs but not in IGFs (arrow, Figure 2A). Phylogenetic tree analysis of echinoderm RGPs showed a distinct clade of the echinoderm RGPs, separated from the echinoderm IGFs (Figure 2B). Two major clades (with 95% bootstrap support) were observed within the RGP group, including sea cucumber RGPs and sea star RGPs, suggesting an evolutionary divergence in accordance with the divergence of holothuroids and asteroids.

### Production of a Recombinant *H. scabra* RGP

**Figure 3:**
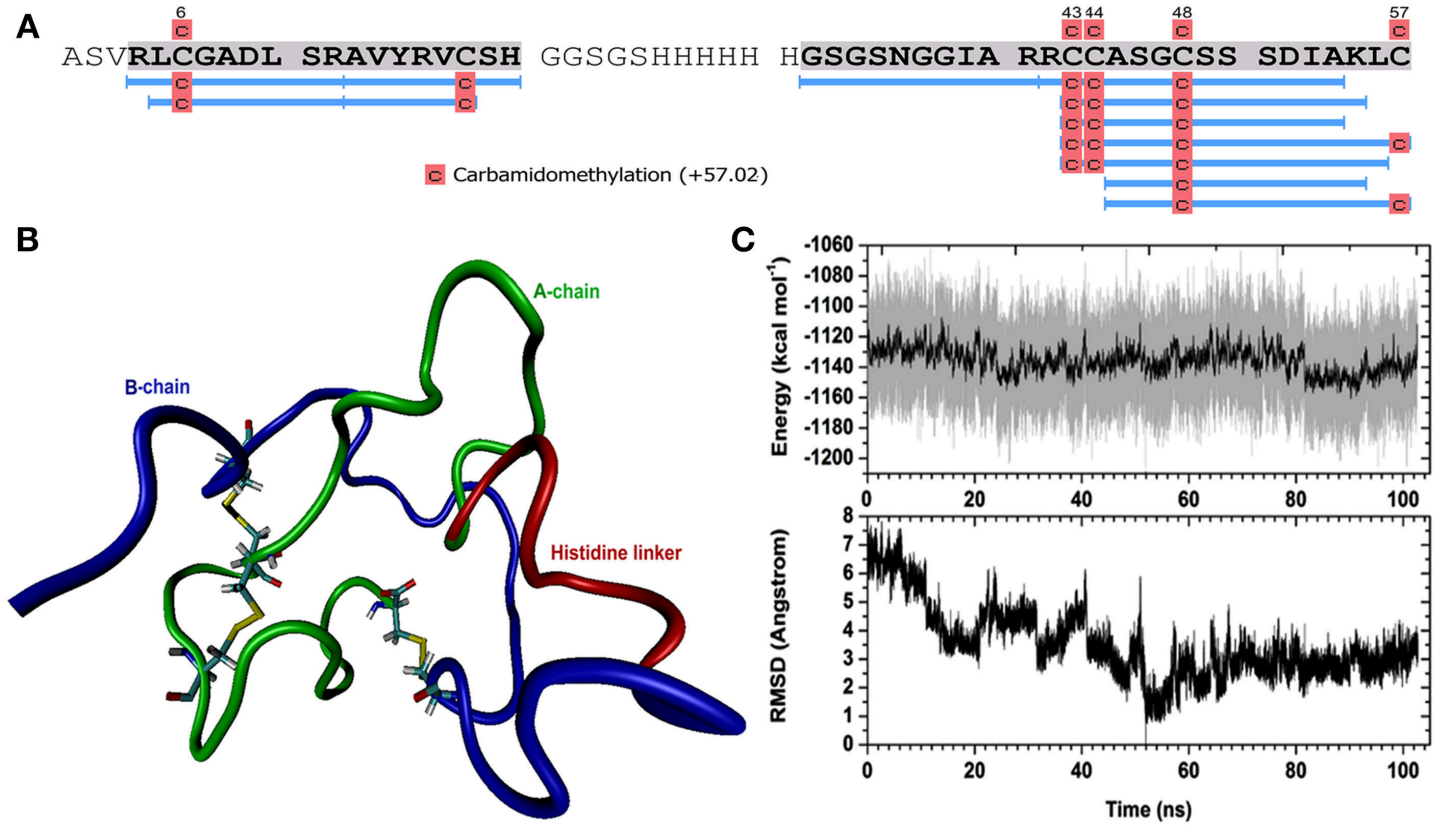
Recombinant *Holothuria scabra* RGP and mass spectrometry (MS) identification. **(A)** RGP sequence showing MS peptide coverage (light blue lines). **(B)** A representative structure of recombinant RGP. Green: A-chain, dark blue: B-chain, red: histidine linker “GSGSHHHHHHGSGS”. **(C)** Upper, potential energy of RGP peptide as a function of time during molecular dynamics (MD). Lower, backbone RMSD (root mean square deviation) during the same MD, compared to the lowest-energy conformation (the representative structure).

A *H. scabra* RGP-pPIC9K recombinant plasmid was successfully electro-transformed into the yeast cells and colonies were grown in different concentrations of Geneticin selective antibiotic (0, 1, and 2 mg/mL) at 30°C for 2 days. Those colonies that showed strong growth, even in high concentration of Geneticin (Supplementary Figure S1), were selected for large-scale RGP production and purification. Mass spectrometry showed the presence of *H. scabra* RGP matching peptides with high confidence (Figure 3A, Supplementary Data S2). MD simulation was used to build a model for the recombinant *H. scabra* RGP and a properly folded 3D structure was obtained (Figure 3B); this model was supported by the relatively stable potential energy (Figure 3C, top) as well as backbone RMSD (root mean square deviation) during the same MD (Figure 3C, bottom).

### *In vitro* GVBD Bioassay

**Figure 4:**
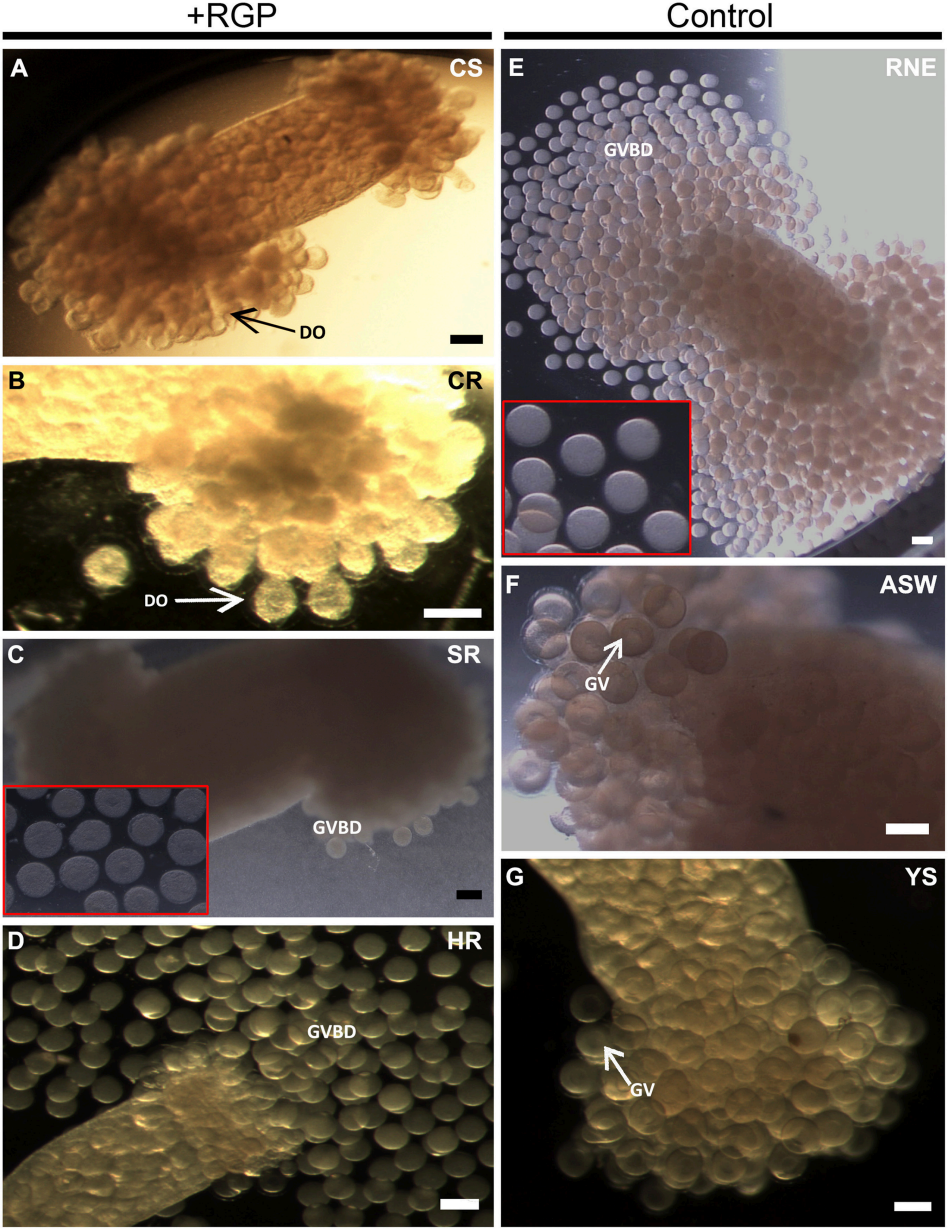
*Holothuria scabra* oocyte maturation bioassay with recombinant RGP. Ovarian segments were incubated for 3 h in **(A)** crude supernatant, CS; **(B)** crude concentrated RGP, CR; **(C)** SepPak-purified RGP, SR; inset shows higher magnification of mature oocytes from ovarian segment incubated in 96 μg/μL SR; **(D)** His and Amicon-purified RGP, HR; **(E)** yeast solution without RGP, YS; **(F)** filtered artificial sea water, FASW; and **(G)** radial nerve extract, RNE. Two insets (red boxes) indicate higher magnification of mature oocytes inside ovarian segment incubated in SR (96 μg/μL) and RNE after 3 h. DO indicates the distorted oocyte; GV, germinal vesicle; GVBD, germinal vesicle breakdown. All scale bars indicate 200 μm.

Recombinant *H. scabra* RGP at four levels of purification was tested for induction of GVBD using ovarian tubules derived from adult *H. scabra* and *H. leucospilota*. Table 2 summarizes the results in both species. GVBD was observed at different efficiencies for the CS, SR and HR treatments, as well as for the positive control using 0.2 μg/μL RNE. CS at a concentration of 1.75–3.50 μg/μL induced GVBD at a relatively low efficiency (6.62–7.57% in *H. scabra* and 0.34–1.39% in *H. leucospilota*), while at 7 μg/μL, no GVBD occurred. Furthermore, oocytes showed a partial distortion at 3.5 μg/μL and complete distortion at 7 μg/μL (distortion is when oocyte shows a rough surface and deformed shape, which is an abnormal phenomenon that usually indicates stressed oocyte) (Figure 4A). CR did not stimulate oocyte GVBD, yet caused oocyte distortion at 56.5 μg/μL, and complete distortion at 113 μg/μL (Figure 4B). GVBD was clearly observed in SR (Figure 4C) and HR (Figure 4D). For SR, almost 100% GVBD occurred at 96 μg/μL, which was comparable to the treatment of 0.008 μg/μL HR. GVBD was observed in both released oocytes and in-tubule oocytes.

**Table 2:** GVBD at 3 h post-incubation with test medium in *Holothuria scabra* and *H. leucospilota*.

**Tested medium**	**Final peptide concentration (μg/μL)**	***H. scabra* % GVBD**	***H. leucospilota* % GVBD**
Crude supernatant (CS)	1.75	6.62 ± 2.15	0.34 ± 0.06
	3.50	7.57^*^± 3.62	1.39^*^± 0.86
	7.00	0^**^	0^**^
Crude concentrated RGP (CR)	28.25	0	0
	56.50	0^*^	0^*^
	113.00	0^**^	0^**^
SepPak-purified RGP (SR)	24.00	2.45 ± 1.70	1.37 ± 0.86
	48.00	65.23 ± 14.58	56.00 ± 17.14
	96.00	99.42 ± 0.37	98.71 ± 0.34
His and Amicon-purified RGP (HR)	0.004	97.09 ± 0.80	86.29 ± 2.62
	0.008	99.31 ± 0.30	89.15 ± 1.76
	0.016	99.30 ± 0.26	93.26 ± 1.82
RNE (0.2 μg/μL)	0.20	96.78 ± 1.58	96.67 ± 3.76
Yeast solution without RGP (YS)	0^a^	0.42 ± 0.38	0
	0^b^	0^*^	0^**^
	0^c^	0^**^	0^**^
Filtered artificial sea water (FASW)	0	0	0

* *Oocytes with partial distortion*.

** *Oocytes with complete distortion*.

a *20 μl YS and 60 μl filtered artificial sea water (FASW)*.

b *40 μl YS and 40 μl FASW*.

c *80 μl YS in total*.

In the positive control group, 0.2 μg/μL RNE could induce 96.78% GVBD in *H. scabra* and 96.67% in *H. leucospilota* (Table 2). The oocytes showed extrusion from ovarian segments and matured in the RNE medium (Figure 4E). In negative controls, neither significant oocyte extrusion nor GVBD was occurred in YS and FASW treatments (Figures 4F,G). However, the YS caused oocyte distortion (Table 2).

### *In vivo* Spawning Bioassay, Fertilization, and Larval Development

**Figure 5:**
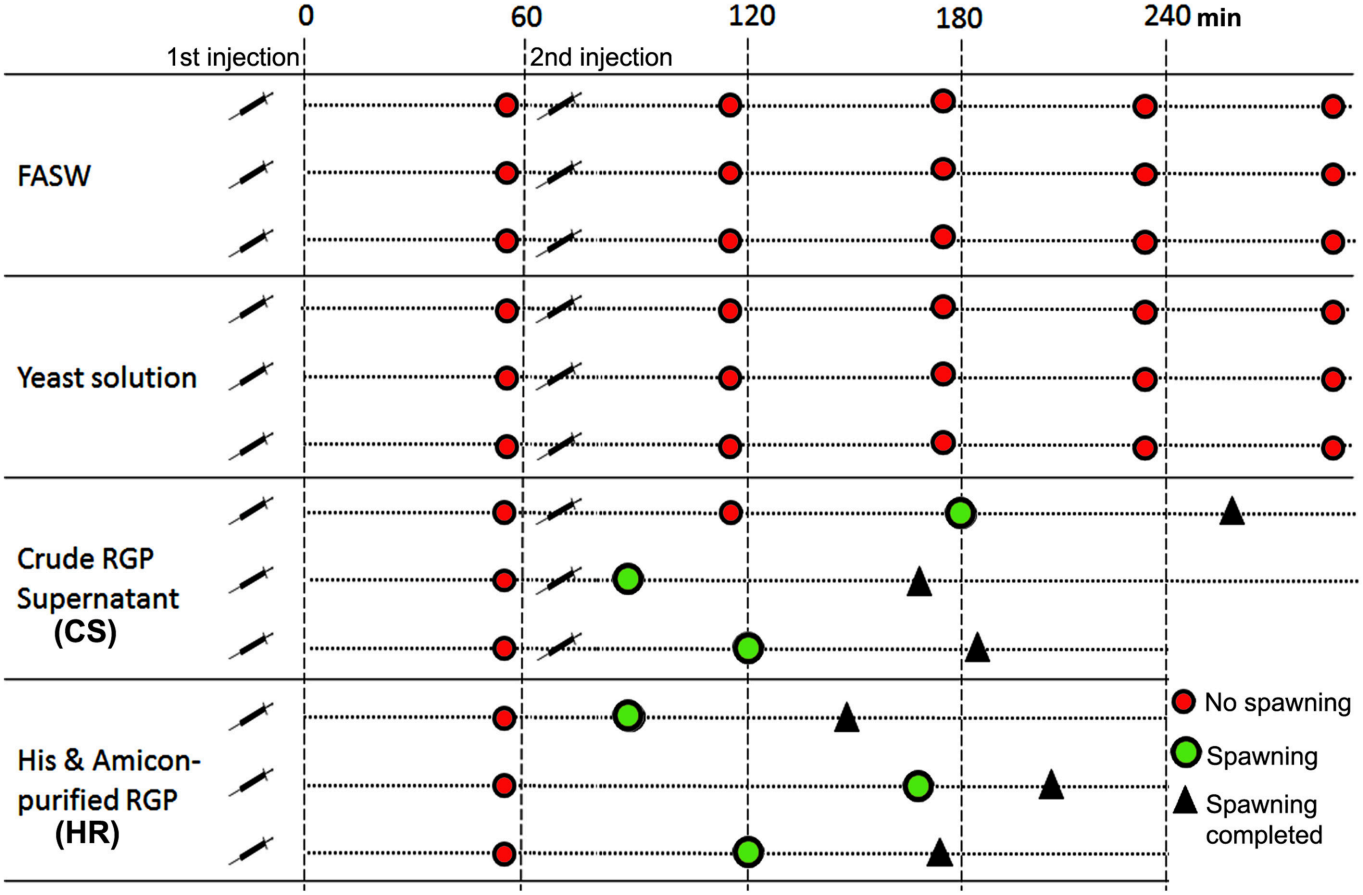
Summary of *Holothuria scabra* spawning bioassay with recombinant RGP. Negative controls included filtered artificial seawater (FASW) and yeast solution without RGP. Test solutions included crude RGP supernatant (CS) and His and Amicon-purified RGP (HR). The first injection was a 2mL volume and the second was a 3mL volume.

**Figure 6:**
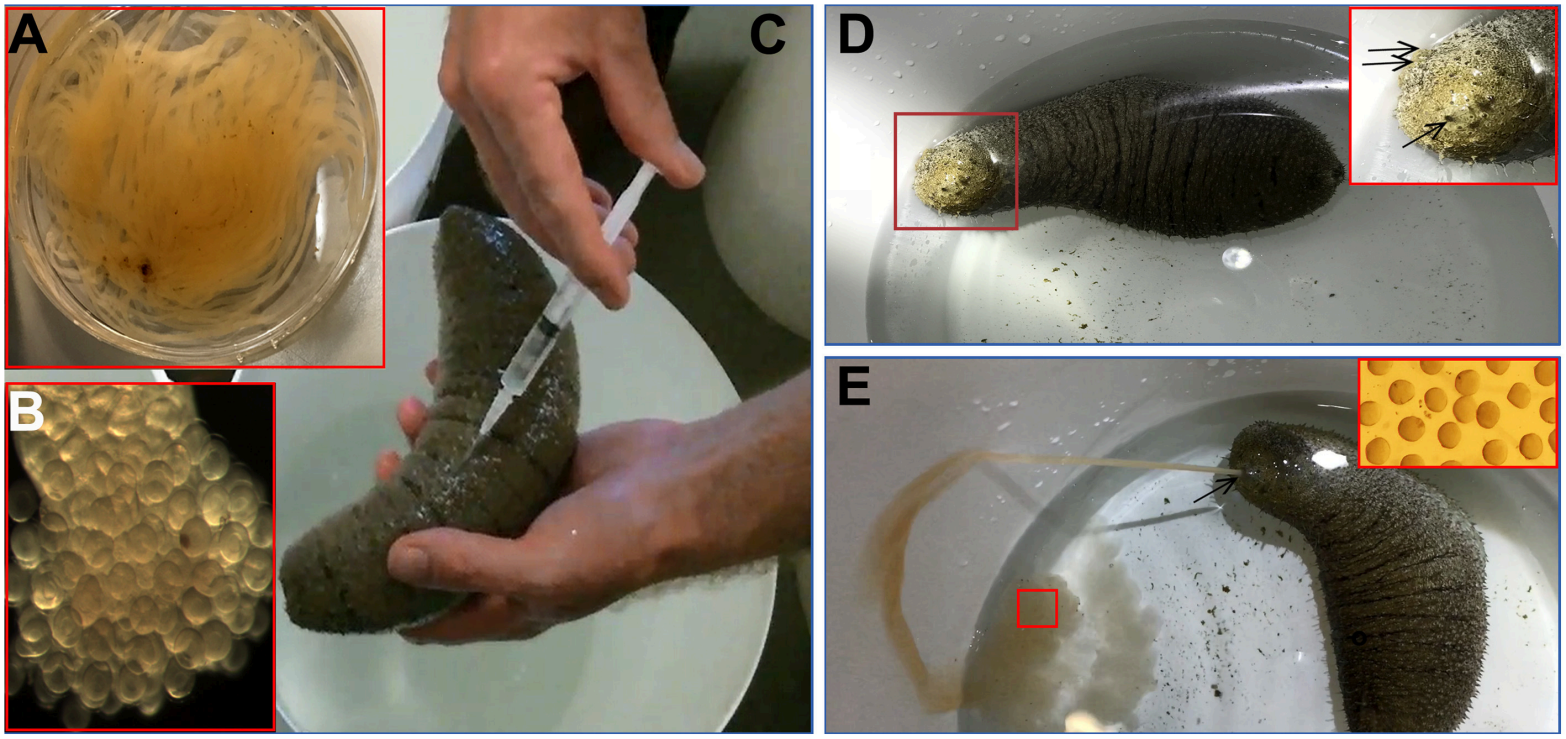
*Holothuria scabra* spawning bioassay with recombinant RGP. **(A)** Total gonad from an individual showing tubules. **(B)** Immature oocytes (170 ± 6.4 μm in diameter) with germinal vesicle before spawning. **(C)** Injection of sample into the body wall. **(D)** Head waving observed prior to spawning. Inset, higher magnification of sea cucumber head (red box), showing the location of gonopore (single arrow) and mouth (double arrow). **(E)** Sea cucumber spawning oocytes from gonopore. Inset, high magnification view of spawned oocytes (red box) indicating they were mature.

Based on results obtained from the GVBD bioassay, the CS and HR were tested in a spawning bioassay in *H. scabra*. As negative controls, FASW and yeast solution were tested. A summary of the results is shown in Figure 5. All animals used in the experiment had developing ovaries, as determined by biopsy (Figure 6A). This was most clear following biopsy and oocyte observation under a light microscope, showing immature oocytes at ≥150 μm diameter (Figure 6B). After injection (Figure 6C), no animals displayed stress-induced evisceration (ejection of internal organs). Head waving was observed in animals injected with CS and HR at 30–60 min post-injection (Figure 6D, Supplementary Movie S1). At this time, the gonopore (Figure 6D, inset single arrow) located anteriorly and opposite the mouth position (Figure 6D, inset double arrow), was clearly visible. For CS, no animals spawned at 60 min but after 2nd injection, spawning started at 90, 120, and 180 min from time 0, respectively (Figure 6E). Spawning was completed within 60–80 min post-initiation. For HR, only one injection was performed (at 40 μg concentration) and spawning occurred at 90, 120, and 170 min from time 0, respectively. All spawned eggs had undergone GVBD and were mature (Figure 6E, inset). Spawning was complete within 30–60 min post-initiation. For both negative controls, which were injected twice with either FASW or yeast solution, no head waving or spawning was observed.

**Figure 7:**
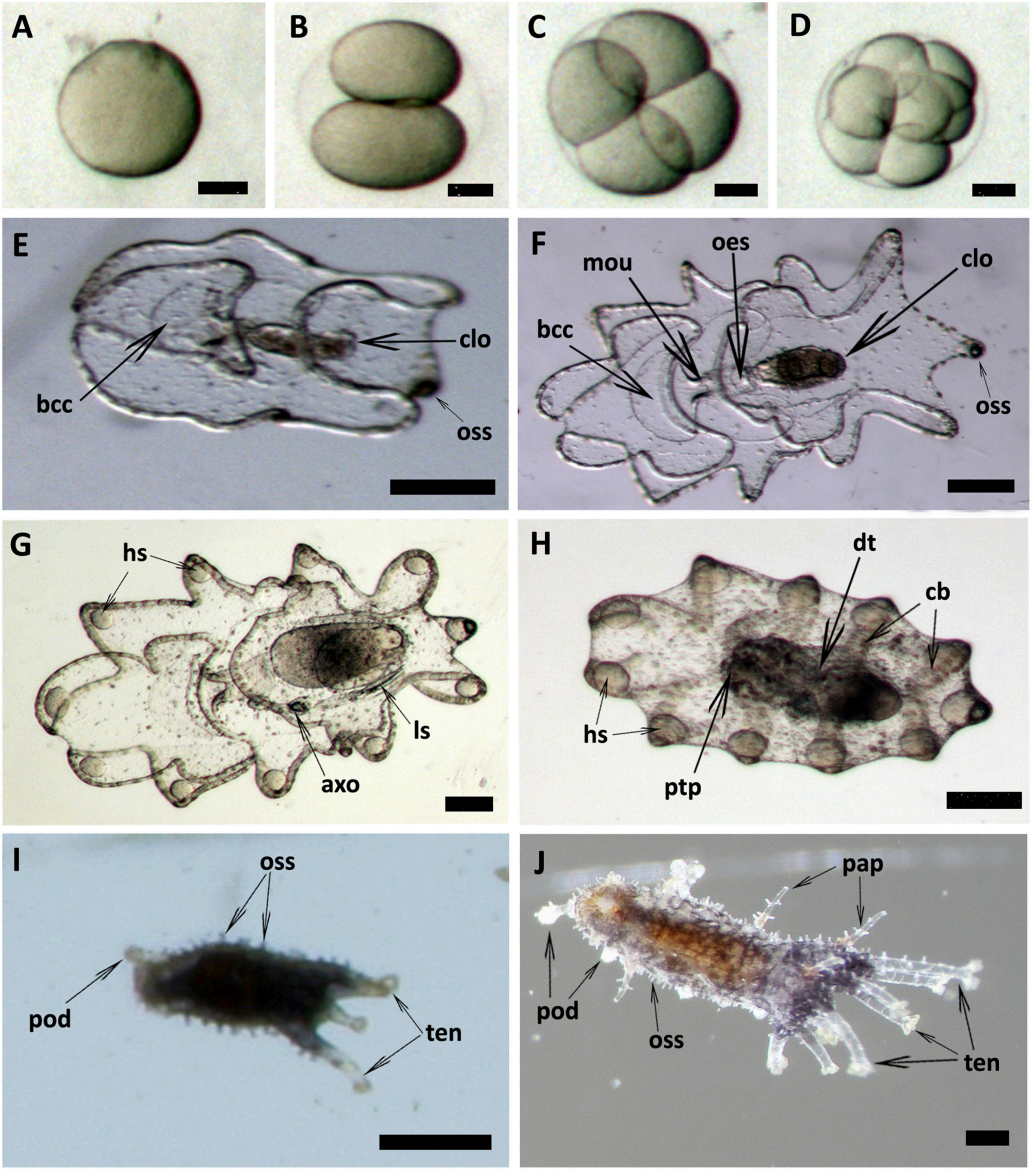
Embryonic, larval development and juvenile growth of *Holothuria scabra* from RGP-matured eggs. **(A)** the matured eggs were fertilized, then **(B–D)** 2-cell, 4-cell and multi-cell division was occurred until 8 h post-fertilization. **(E)** early auricularia stage at 2 days post-fertilization (dpf). Buccal ciliated cavity (bcc), cloaca (clo), ossicle (oss). **(F)** Mid-auricularia stage at 5 dpf. Mouth (mou), oesophagus (oes). **(G)** Late-auricularia at 14 dpf. Hyaline sphere (hs), axohydrocoel (axo), left somatocoel (ls). **(H)** Doliolaria at 16-day; digestive tract (dt), ciliary band (cb), primary tentacles primordium (ptp). **(I)** Pentactula at 21 dpf. Tentacle (ten), podia (pod). **(J)** Juvenile at 36 dpf. Papillae (pap). Scale bars in **(A–D)** are 50 μm. Scale bars in **(E–H)** are 100 μm. Scale bars in **(I,J)** are 500 μm.

Spawned oocytes were taken for fertilization with conspecific sperm, then cultured for larval and juvenile development (Figure 7, Table 3, Supplementary Movie S1). The development of embryos, larvae, and juveniles was asynchronous, with different stages and sizes observed simultaneously in culture. Fertilized eggs reached a size of 165–185 μm prior to division into the 2-cell, 4-cell and multi-cellular stages (Figures 7A–D). Embryo size did not significantly change within the period of 1–8 h post-fertilization (hpf). The gastrula could be observed after 8–24 h post-fertilization and reached a size of 210-300 μm (Table 3). At 1 day post-fertilization (dpf), the early auricularia stage was reached (ca. 300 μm) and then from 1 to 3 dpf (300–500 μm) the buccal ciliated cavity, cloaca and ossicle was observed (Figure 7E). At the mid-auricularia stage (3–9 dpf), larval size was notably increased, while the mouth and esophagus were additionally visible (Figure 7F). The late-auricularia, recognizable by the presence of a hyaline sphere, axohydrocoel, and left somatocoel, was observed at 10–16 dpf (Figure 7G) and the larvae were in the 900–1,200 μm in size range. From then, the auricularia started decreasing in size (500–800 μm) and subsequently transformed into the doliolaria stage (Figure 7H). At this stage, primary tentacles primordium, digestive tract and ciliary band were observed. The pentactula was identified at 18 dpf, at which the ossicle, tentacles and podia were formed (Figure 7I). At the pentactula stage, larvae swam close to substrates and settled. Subsequently, podia developed in quantity along the ventral body wall and papillae on the dorsal body wall. Finally, pentactula transformed into juveniles and could be observed at 21 dpf. Juvenile reached a size of 4–5 mm by 36 dpf (Table 3, Figure 7J).

**Table 3:** Summary of development in *Holothuria scabra* embryo, larvae and juvenile stages.

**Stages**	**Time**	**Size (μm)**
Fertilization	0 min	165–185
2-cell embryo	50–70 min	165–185
4-cell embryo	70–120 min	165–185
Multi-cell embryo	2–8 h	185–210
Gastrula	8–24 h	210–300
Early auricularia	1–3 d	300–500
Mid auricularia	3–9 d	500–900
Late auricularia	10–16 d	900–1,200
Dolillaria	16–18 d	500–800
Pentactula	18–21 d	750–1,100
Juvenile	21–36 d	1,100–4,500

## Discussion

The enhancement of aquaculture animal breeding using molecular approaches is now a common avenue for replacing or complementing traditional approaches. This includes the use of neuropeptide/hormone manipulation to enhance breeding strategies. However, this approach requires an in-depth knowledge of the relevant molecular machinery. In sea stars, it is well-established that RGP plays an important role in oocyte maturation and spawning (Mita et al., 2009; Mita, 2013, 2016) and we speculated that it could also play a similar role for sea cucumbers. In this study, we have produced a recombinant sea cucumber RGP using a yeast expression system and its bioactivity was confirmed through stimulation of oocyte maturation and spawning.

In the echinoderms, RGP was first characterized in *Patiria pectinifera* (called PpeRGP) and then identified in other sea star species, including *Asterias amurensis* RGP (AamRGP) and *Aphelasterias japonica* RGP (AjaRGP). The protein structure of AamRGP appears to be closer to AjaRGP than it is to PpeRGP (Mita, 2016). Cross-species *in vitro* oocyte maturation experiments showed that both AamRGP and AjaRGP could not induce oocyte maturation and ovulation in the ovary of *P. pectinifera*, although the PpeRGP was active in ovaries of *A. amurensis* and *A. japonica* (Mita, 2016). To further investigate the conservation of RGP in echinoderms, in this study we explored the holothurian RGP and performed sequence comparisons and phylogenetic analysis. The *Holothuria* RGP showed highest homology to other echinoderm RGPs, with the highest conservation of amino acid composition found among the various *Holothuria* species (~87%), suggesting possible cross-species bioactivity for RGP activity within *Holothuria* species, although further functional assays were required to confirm this. All RGPs contain 6 highly conserved cysteine residues, 4 and 2 of which are present in the A- and B-chains, respectively. This indicates a high selective pressure during the evolution of these critical cysteine residues, which are responsible for disulphide bridge formation. Despite the observed conservation within the A- and B-chains of echinoderm RGPs, the amino acid composition within the RGP-related peptide region was considerably variable. RGP belongs to the insulin/IGF/relaxin family, which include two sub-families such as insulin/IGF and RGP families (Wilkinson and Bathgate, 2007). Our alignment also showed conservation of amino acid composition, especially within the putative A- and B-chain regions, among RGPs and IGFs in echinoderms. The major characteristics of RGP that are different to IGF include: (i) the conservation of a glycine residue prior to the third conserved cysteine residue with the A-chain of RGPs, but not IGFs; and (ii) an amino acid at the C-terminal end of the A-chain, which is the cysteine in RGPs but asparagine in IGFs. The phylogenetic analysis also confirmed a common root for these two peptide families. Within the RGP clades, two distinct sister clades were observed for RGPs from two different echinoderm classes, including the sea cucumber (holothuroids) RGPs and the sea star (asteroids) RGPs (see Figure 2B), which reflects the evolutionary divergence of this peptide during the evolution of echinoderms.

Following protein expression and purification, an average of 0.7 mg pure RGP was obtained from 1 L of crude supernatant. This yield is comparable with previous studies, including the purification of FSH and LH recombinant proteins by using the same purification technique. For example, 0.4–0.9 mg/L recombinant yellowtail kingfish FSH (Sanchis-Benlloch et al., 2017) and 2.5 mg/L for the grouper FSH (Chen et al., 2012). Lower yields have been reported, such as 0.1 mg/L for the tilapia FSH (Aizen et al., 2007), 0.3 mg/L for the carp LH (Hollander-Cohen et al., 2017) and 0.08 mg/L for the tilapia LH (Kasuto and Levavi-Sivan, 2005). Generally, the yield was suggested to be varied depending on culture conditions (Yu et al., 2010) or the formation of dimeric forms (Aizen et al., 2007). The yield of the heterodimer was usually significantly lower than that of the monomer probably because of the complex structure of the former, for example, 6.5 mg/L were produced for the tilapia FSHβ (monomer), while a yield of only 0.1 mg/L was obtained for the tilapia FSHβα (heterodimer) (Aizen et al., 2007). Production of the recombinant *H. scabra* RGP in the yeast expression system is an effective approach to obtain the copious amounts of RGP that would be required for commercial applications. However, the process of obtaining the slightly cheaper partially purified RGP and delivering to animals on a mass scale still requires optimisation and development of a method that is less laborious.

To confirm the successful production of the recombinant *H. scabra* RGP, in-solution digestion followed by LC-MS/MS was performed, which provided evidence of almost complete sequence coverage (see Figure 3A). The spectra additionally suggested the carbamidomethylation on four cysteines, which was potentially the result of reduction on cysteine residues and/or disulphide bridges. No other post-translational modifications were detected for the recombinant RGP. The MD simulation suggested the formation of three disulphide bonds and a disulphide bridge is formed within the A-chain. In terms of secondary structure, there was mainly turn and coil structures formed during the simulation, such as helix and β sheet, which could also be seen from the RMSD calculation that shows significant fluctuation with respect to time. In conclusion, we found that the expression and purification of the recombinant RGP in our study was successful in term of quantity and a properly folded structure as confirmed by MD simulation. Therefore, this established RGP expression and purification procedures that could be applied and/or used as a fundamental procedure for RGP mass production for future uses.

*In vitro* and *in vivo* bioassays were used to assess the bioactivity and function of recombinant RGP in the sea cucumbers, *H. scabra* and *H. leucospilota*. The *in vitro* GVBD bioassay demonstrated that our recombinant RGP could induce GVBD, even at low concentrations (see Table 2). The recombinant *H. scabra* RGP peptide (SR, HR) could induce oocyte maturation/GVBD in both *H. scabra* and *H. leucospilota*, which is consistent with the conservation and similarity in the amino acids of RGP precursor (see Figure 2). Hence, a “generic” RGP recombinant may be employed to induce GVBD and spawning in more than one species of sea cucumber, although additional experiments showing compatibility of our RGP recombinant with other commercially important sea cucumber species should be verified in the future. Similarly in previous work, synthetically produced RGP could successfully induce GVBD in the sea stars *Asterias aumerensis* and *Asterina pectinifera* using *in vitro* bioassays (Kanatani et al., 1971; Mita, 1993). However, individual synthetic chains of RGP (either A- or B-chain) did not induce GVBD or spawning, whereas a synthetic heterodimeric form could induce GVBD and ovulation within 1 h of incubation in the Crown-of-Thorns sea star, *Acanthaster planci* (Mita et al., 2015). We found that partial GVBD and distortion of oocytes occurred when ovarian fragments were incubated with the crude supernatant CS and CR (see Table 2, Figure 4), which was likely due to a high salt concentration within these crude extracts. Meanwhile, a lower efficiency of GVBD was observed in the partially purified RGP (SR). Therefore, we suggest that it is essential to perform a complete or partial purification of the recombinant RGP before being employed as an oocyte maturation stimulant, in order to maximize the mature oocyte quality and quantity for further fertilization.

A recombinant RGP has advantages over the use of crude RNE, since it does not require the sacrifice of sea cucumbers, and the potential for other RNE factors that may stress the animals. Our bioassay experiments found that RGP stimulation of spawning did not cause evisceration or any observable stress to broodstock. In contrast, several traditional approaches in spawning stimulation [e.g., thermal shock, drying, and water pressure, sperm-induced spawning, dried alga spirulina bathing treatments (Battaglene et al., 2002; Al Rashdi et al., 2012)], could lead to significant animal stress. Another important factor that needs to be considered is the sea cucumber gonadosomatic growth peaks during the year (Battaglene et al., 2002). The traditional approaches usually require sea cucumbers at fully mature gonad stage (gonadal stage IV) for efficient spawning, but we showed in the current study that RGP could induce spawning as early as gonadal stage III (gonad weight 30–50 g and oocyte diameter ≥150 μm). The receptor for RGP still remains elusive, although in sea stars it has been recognized that the RGP is a ligand to a G protein-coupled receptor that is expressed on follicle cells of growing to fully growing ovaries (stages III to V) (Mita et al., 2011a,b). Thus, we hypothesize that follicle cells and gonadal stages could affect RGP efficiency in sea cucumbers, depending on the presence of an RGP receptor.

*In vivo* spawning by injection of RGP in *H. scabra* was successfully achieved using pure RGP (HR), showing that the sea cucumbers exhibited the head waving characteristic at 30–60 min post-injection, and subsequently spawned at either 90, 120, or 170 min post-injection (see Figure 5). Furthermore, spawning was complete within 30–60 min with no subsequent additional spawning observed. This novel method is superior to other traditional stimulating methods since it does not require several days of pre-stimulation, such as thermal stimulation, prior to a spawning event. In addition, the use of RGP is a time and cost-effective procedure since it allows a rapid and highly controllable spawning event.

Injection with the unpurified crude RGP (CS) resulted in no spawning in the first 60 min post-injection, and as such a second injection was performed, leading to spawning within 30, 60, or 120 min following the secondary injection. Spawning after the second injection was completed within 60–80 min. However, we observed that the animals did not show pre-spawning head waving behavior. Hence, we cannot preclude that stress induced spawning might occur when the animals were subjected to high concentration of RGP solution from 2 injections. These results support the observations from the GVBD *in vitro* assay in which the oocytes treated with high concentration of crude RGP were deformed. Therefore, we suggest that not only purity but also the concentration of RGP is critical and are factors for maximum success for fertilization in artificial breeding of sea cucumbers. Further studies are required to determine the minimum recombinant RGP threshold/concentration for efficient spawning induction in the sea cucumbers.

In this study, we found that the development of the *H. scabra* larvae produced from RGP-induced spawning proceeded normally and was comparable to previous studies engaged the traditional spawning stimulations for fertilization and larval production (Battaglene et al., 1999; Mercier et al., 2000; Ivy and Giraspy, 2006; Al Rashdi et al., 2012; Ajith Kumara et al., 2013; Robinson et al., 2013; Abidin et al., 2016). For example, the pentacula stage larvae were predominant at 18-21 dpf (see Table 3), which is similar to the previous reported durations of 13–26 dpf of metamorphosis to pentaculae (James, 2004; Dabbagh and Sedaghat, 2012; Mazlan and Hashim, 2015; Abidin et al., 2016). Furthermore, the larvae were reared through to their juvenile stage and appeared normal in their development. This result demonstrates that recombinant RGP for induced spawning is applicable in artificial breeding and that there are potential benefits for its use in sea cucumber hatcheries. However, the survival rates from fertilized oocytes, using the recombinant RGP, until the juvenile stages, need to be studied further.

## Conclusions

This is the first study to demonstrate the production and use of a recombinant RGP in oocyte maturation and spawning induction in sea cucumbers. The outcome of this study provides a breakthrough new approach to artificial breeding in the sea cucumbers by using a recombinant RGP. We have developed the experimental procedures for the production of the recombinant RGP that could successfully induce *in vitro* oocyte maturation as well as spawning activity in sea cucumber broodstock. However, in order to help with hatchery production, further investigations are required in the future in order to obtain the optimal concentration and practicable delivery method. Furthermore, to understand the mechanism of RGP bioactivity in sea cucumbers, molecular signaling pathway analysis should be implemented. This information could help to overcome the seasonality requirements for RGP induced spawning.

## Ethics Statement

The collection and handling of the animals in this study was carried out in accordance with the guidelines for the care and use of laboratory animals at the University of the Sunshine Coast, Australia.

## Author Contributions

SC and AE conceived the idea and provided oversight of the project from beginning to the final manuscript. HC carried out the laboratory components, *in vitro* and *in vivo* experiments and manuscript writing. LT carried out the *in vivo* spawning assay and was lead to the larvae and juvenile rearing at the Darwin Aquaculture Centre. MS, JN, and PP contributed to the sub-cloning, cloning, and all the steps in the laboratory production of the recombinant RGP. TW performed MS and molecular dynamic simulation. SS constructed sequence alignments and phylogeny analysis images. All authors provided critical feedback and contributed to the writing and review of the final manuscript.

### Conflict of Interest Statement

The authors declare that the research was conducted in the absence of any commercial or financial relationships that could be construed as a potential conflict of interest.

## Supplementary Material

Supplementary Data S1 Amino acid sequences of relaxin/insulin-like peptides in echinoderms.

Supplementary Data S2 Mass spectrometry (MS) data.

Supplementary Figure S1 Antibiotic screening for high expression of RGP in yeast colonies.

Supplementary Movie S1 A combinative movie of RGP injection, head waving behavior, spawning response, and larvae and juvenile development.
