# Supplementary material for: Aquaculture Breeding Enhancement: Maturation and Spawning in Sea Cucumbers Using a Recombinant Relaxin-Like Gonad-Stimulating Peptide
Source: Front Genet. 2019 Feb 19;10:77. doi: 10.3389/fgene.2019.00077 (PMC6389678; doi:10.3389/fgene.2019.00077)
Supplement: Supplementary Data S1 — Amino acid sequences of relaxin/insulin-like peptides in echinoderms. [file Data_Sheet_1.docx]

Supplementary Data S1: Amino acid sequences of relaxin/insulin-like peptides in echinoderms

Article Title:

“Aquaculture breeding enhancement: Maturation and spawning in sea cucumbers using a recombinant relaxin-like gonad-stimulating peptide”

***Authors:***

Hoang Dinh Chieu^1,2^, Luke Turner^3^, Meaghan K. Smith^1^, Tianfang Wang^1^, Josephine Nocillado^1^, Peter Palma^1, 4^, Saowaros Suwansa-ard^1^, Abigail Elizur^1^, and Scott F. Cummins^1,*^.

***Institutional affiliation:***

^1^ Genecology Research Centre, University of the Sunshine Coast, 90 Sippy Downs Drive, Sippy Downs, Queensland 4556, Australia;

^2^ Research Institute for Marine Fisheries (RIMF), 224 LeLai Street, HaiPhong City, Vietnam;

^3^ Tasmanian Seafoods Pty. Ltd., Tasmania, Australia;

^4^ Aquaculture Department, Southeast Asian Fisheries Development Center, Tigbauan, Iloilo 5021, Philippines.

***Address correspondence to:***

* Assoc. Prof. Scott F. Cummins. Genecology Research Centre, Faculty of Science, Health, Education and Engineering, University of the Sunshine Coast, Australia. Tel: +61 7 5456 5501; Fax: +61 7 5456 5010; email: scummins@usc.edu.au

**Abbreviations:**

RGP: Relaxin-like Gonad-stimulating Peptide

IGF: Insulin-like Growth Factor

Hle: *Holothuria leucospilota* (Brandt, 1835)

Hsc: *Holothuria scabra* (Jaeger, 1833)

Hgl: *Holothuria glaberrima* (Selenka, 1867)

Apj: *Apostichopus japonicus* (Selenka, 1867)

Aru: *Asterias rubens* (Linnaeus, 1758)

Aam: *Asterias amurensis* (Lutken, 1871)

Aja: *Aphelasterias japonica* (Bell, 1881)

Apl: *Acanthaster planci* (Linnaeus, 1758)

Ppe: *Patiria pectinifera* (Muller & Troschel, 1842)

Spu: *Strongylocentrotus purpuratus* (Stimpson, 1857)

**Amino acid sequences of RGP/IGF peptides in echinoderms include:**

>Hle-RGP

MASKTIRVVFFAAVCVLLVLEEAASTRLCGRELSRAIYRICSHGKRGYPMVDLEEEDFSQELDTEWDEFLAQALTGLLESRTFAADIESDRYFTIPQRFRRSGGIARRCCASGCSSSDIAKLC

>Hsc-RGP

MASKTTRVVFFAAVCVLLVLEHAASVRLCGADLSRAVYRVCSHGKRGYPMIDIEEDDFSQELDTELDEYLAQALTGFLESRSFAADIESDRYYTIPQRFRRNGGIARRCCASGCSSSDIAKLC

>Hgl-RGP

MASKATRVVFFAAVCVLLVLEEAASVRLCGADLSRAVYRVCSHGKRGYPMVDLEEEDFSQELDTEVDEFLAQALTGFLASRSFAADMESDRYYTLPQRFRRNARGGIARRCCASGCSSSDIAKLC

>Apj-RGP

MTTQSTTVIILRIFCVLIAVREAASIRLCGPDLSRAVYQICSHGKRGYIPPTFNSEDDQLNQEFGTDLEEYLAETIKEYLKPNSLYDDVERELYPSLPRGFRRVTRTGGIARRCCSTGCSSSDIAKLC

>Aru-RGP1

MANYRLILEATCLLVLLINTALYAEAAEKYCDEDFHMAVYRTCTEHKRSGRSAFSLNDFFRSNSKRTAGSPRPDDDFFLTMQKRPETYVGMGSYCCLVGCTRDQLSQVC

>Aru-RGP2

MTSCSHQMLALLSAVYILIFFLGGLPAVHARSDHASVKHFCGLEFSYAVVTACGEAKRSIRSAPFFDMFPVFKSPERIPADFDDSSMIHVRKRQDYQGMATYCCTNGCTISQLTNSGIC

>Aam-RGP

MANYRLILEATCLIVLLINTALYAEAAEKYCDEDFHMAVYRTCTEHKRSGRSAFSLNDFFRSNSKRTAGSPRQDDDFFLTMQKRPETYVGMGSYCCLVGCTRDQLSQVC

>Aja-RGP

MANHRLILEATCLLVLLINTALYAEAAPKYCDEDFHMAVYRTCSEHKRSGRSTYSLNDLLTLNRLRSNPKRTVGSLEDDDLYLTMQKRTETYVGMGSYCCTVGCTREELSQVC

>Apl-RGP

MANNLRRRFQATCLALLILQATINTGAVGEKFCDNDFHLAVYQTCSTHKRGDGEPVLSLKDVLTGSRLRGNIKRSFGSTLEDEAFFASRLVKRSEYDGIASYCCIHGCTPSELAVVC

>Ppe-RGP

MTSNNRHLFQATCLVLLLLHAAFHGGALGEKYCDDDFHMAVFRTCAVSKRSQPGMSLSDVLTMNRFRGHNIKRSIDSTLEDNAFFMSGLEKRSEYSGIASYCCLHGCTPSELSVVC

>Spu-IGF1_SPU_007203.3

MVCFRYPVAVMSVVLLALLRHVTASFPLLCGQELVKAVAAVCNDRGYYGQPSKRSAGIFELETRAKTFLKSGMSRGETRRSKRGARTGLIVTECCLNRCSVSHLESYCNPLPPDAVHDAEVHIRLEKSAEEDADEGRPQDGPSQLDTATGTVPETDTSETRGRVRIDAVEKVISERLIPTSTTGSSPSPSRKKPRKDKSERRNSSREAKQARREERRRNRERGSGGRSRSGRRKDKDNDRASRAKRHGLNLWRNMFSDKFFSDIPGLENQPNLHPVNGRAPSSTTIDTFQMKSSIPIQDSPEGNGKENFEQSSINQEADKKMRFSALMTKLRTMVLKFPKDR*

>Spu-IGF2_SPU_030139.3

MDPFRVLLYMVTFLLYVVGPISSFRLCGRELADALAVVCKGRGYYIDDSEIAQKDSPIVPHHVASSFLGSSSASAHSRQRRRVRTGQIVNECCDKECSNNIMESYCNRRTPEVPPESAISENPSEEITEDSTLRTDGESTEIRTDTNPATNLEVPSPDANTPDATATSDVEQPRSDNTTAVEKPRKKDNGKGKNSSLESSTKKNRTSKGMSKEDRRRIASDERRASRERKKELSRERRKRLKLQQRKDKKKKKRLESAERNRGTDHMGLSEDSTLLAREPLGIDVRKRFHHTPRSSREQASTATHALDDDPATSRQERRRTQSRPSSRERKTHRTTTATAREEEMQRERRNVMQRLTGLFL*

>Aru-IGF1_ ALJ99972.1

MFRNTSTMRALLLLDVIFVALVLPITAWPKICGEQLVETVSLVCSTRGFYSHRDSKRDVEVFQNERAAKSFLGSRIGSRQRRRTGRIATECCDRICSFDIVESYCNPWPVAIESRDPPLSPVAPGRVREDKSADVDYMYNPDVVDVEEANSVIQREEDLIDDIETQEQEIEQDEEQNMQTLPEEDAEDTDIREPEDVEESFPVPVPTKKRRKVEGRRSKESKNKGGKSEGKNKKRSGSREGGRSSRRSRGKSSRSKKQRDGRERSKRWEGLDTSHPVKEPTARSVLGRVDTRPFRNFLYNRYTVDEKRDTERESYRAVAPLTGYNSHRGGSQPDNHPTLAALYNLAVKLA KGLQH

>Aru-IGF2_ALJ99973.1

MNQYQLIVLFEVLAHASMLNYASPVQLCGRELTETLRSICGDRGYYSPGQSFSRRAPTHDGIATRCCQSLCESSILETYCNLPAPPSQTQPSTAAPTTTTKMAPLTEDRRTKDVVVDYSDQLATEGSQMSRVDGVLTHDTVTNRSKTTTESNEGSYDNEEGAPYDKPDDSSPSERGESIQDEDNEVNKPEPNNIRDNSKERGRNRTHKGVSSERRANNSRRRGLSSERRGSSSSRREEKLRRRRQRHRERELREQRKQSNSKRKSKGDKKDHSVAATTPLAVQERPLKNGGRNSTSGEHSSVNGTETDTAGAGSPEVKKDDLITTITAVLSDMIGFQPDNGNR

>Hgl-IGF

MHLNQMCKILVYLSLSLVSMVLSADGTGQKYCGEALLEALAFICGDRGYYGMTSGIHGRSVSRSPFLSEERANSFLTNDGTRNRRGTGRIVTECCENYCTTSVLESYCNFATELPTELSTERTTTEPSASPRRNSRHAGADITPDGETPRQSNNGRNRGNPNRHSEDNPEDNIDETVTHRTETEDQSRSNENRGNRGKGNRDNQNNCRNSKKKGSKKKGNRKCRPGNEDSASEDGGNRRPPSAATATEGADRSSGSRGGRGSNHQTGRGSDRGSSRSNGGKGKGNRNQDRNQTDAGADITPDGETPRQSNNGRNR
