# Supplementary material for: Aquaculture Breeding Enhancement: Maturation and Spawning in Sea Cucumbers Using a Recombinant Relaxin-Like Gonad-Stimulating Peptide
Source: Front Genet. 2019 Feb 19;10:77. doi: 10.3389/fgene.2019.00077 (PMC6389678; doi:10.3389/fgene.2019.00077)
Supplement: Supplementary Data S2 — Mass spectrometry (MS) data. [file Data_Sheet_2.docx]

Supplementary Data S2: Mass spectrometry (MS) data

Article Title:

“Aquaculture breeding enhancement: Maturation and spawning in sea cucumbers using a recombinant relaxin-like gonad-stimulating peptide”

***Authors:***

Hoang Dinh Chieu^1,2^, Luke Turner^3^, Meaghan K. Smith^1^, Tianfang Wang^1^, Josephine Nocillado^1^, Peter Palma^1, 4^, Saowaros Suwansa-ard^1^, Abigail Elizur^1^, and Scott F. Cummins^1,*^.

***Institutional affiliation:***

^1^ Genecology Research Centre, University of the Sunshine Coast, 90 Sippy Downs Drive, Sippy Downs, Queensland 4556, Australia;

^2^ Research Institute for Marine Fisheries (RIMF), 224 LeLai Street, HaiPhong City, Vietnam;

^3^ Tasmanian Seafoods Pty. Ltd., Tasmania, Australia;

^4^ Aquaculture Department, Southeast Asian Fisheries Development Center, Tigbauan, Iloilo 5021, Philippines.

***Address correspondence to:***

* Assoc. Prof. Scott F. Cummins. Genecology Research Centre, Faculty of Science, Health, Education and Engineering, University of the Sunshine Coast, Australia. Tel: +61 7 5456 5501; Fax: +61 7 5456 5010; email: [scummins@usc.edu.au](mailto:scummins@usc.edu.au)

**2.1. *Holothuria scabra* RGP Precusor**

[grey, signal peptide; yellow, mature peptide; red, cleavage site; green, amide donor for C-terminal amidation; blue, cysteine residue possibly for disulfide bridge formation; *, full sequence]

>Hsc_RGP

MASKTTRVVFFAAVCVLLVLEHAASVRLCGADLSRAVYRVCSHGKRGYPMIDIEEDDFSQELDTELDEYLAQALTGFLESRSFAADIESDRYYTIPQRFRRNGGIARRCCASGCSSSDIAKLC*

**2.2. Table data in MS**

**All the peptides were found in MS matched with RGP construct as the following table:**

| Protein Accession | Peptide | Unique | -10lgP | Mass | ppm | m/z | z | RT | Scan | #Spec | Start | End | PTM |
| --- | --- | --- | --- | --- | --- | --- | --- | --- | --- | --- | --- | --- | --- |
| GSS_seacucumber | R.CCASGCSSSDIAKLC | N | 123.82 | 1674.647 | -4.3 | 838.3271 | 2 | 4 | 568 | 1 | 43 | 57 | Carbamidomethylation |
| GSS_seacucumber | R.CCASGCSSSDIAK.L | N | 118.98 | 1401.532 | -6.6 | 701.7687 | 2 | 1.6 | 208 | 1 | 43 | 55 | Carbamidomethylation |
| GSS_seacucumber | R.LCGADLSR.A | N | 68.27 | 890.428 | -4.7 | 446.2192 | 2 | 3.1 | 405 | 1 | 5 | 12 | Carbamidomethylation |
| GSS_seacucumber | C.ASGCSSSDIAK.L | N | 66.26 | 1081.471 | -6.8 | 541.7391 | 2 | 1.4 | 188 | 1 | 45 | 55 | Carbamidomethylation |
| GSS_seacucumber | R.CCASGCSSSDIAKL.C | N | 59.28 | 1514.616 | -6.4 | 758.3106 | 2 | 4.2 | 608 | 1 | 43 | 56 | Carbamidomethylation |
| GSS_seacucumber | H.GSGSNGGIAR.R | Y | 58.88 | 874.4257 | 30.2 | 438.2333 | 2 | 1.2 | 161 | 1 | 32 | 41 |  |
| GSS_seacucumber | R.CCASGCSSSDIA.K | N | 44.75 | 1273.437 | -4.1 | 637.7233 | 2 | 2.7 | 355 | 1 | 43 | 54 | Carbamidomethylation |
| GSS_seacucumber | R.AVYRVCSH.G | N | 41.54 | 990.4705 | -1.9 | 496.2416 | 2 | 2 | 259 | 1 | 13 | 20 | Carbamidomethylation |
| GSS_seacucumber | C.ASGCSSSDIAKLC | N | 31.91 | 1354.586 | -4.3 | 678.2972 | 2 | 3.8 | 529 | 1 | 45 | 57 | Carbamidomethylation |
| GSS_seacucumber | V.RLCGADLSR.A | N | 30.02 | 1046.529 | -1.8 | 524.2709 | 2 | 2.6 | 351 | 1 | 4 | 12 | Carbamidomethylation |
| GSS_seacucumber | R.AVYRVC.S | N | 29.03 | 766.3796 | -3 | 384.1959 | 2 | 2.9 | 381 | 1 | 13 | 18 | Carbamidomethylation |

**2.3. Diagram of peptides matched with RGP construct in MS**

**Peptide CCASGCSSSDIAKLC:** m/z 838.3271


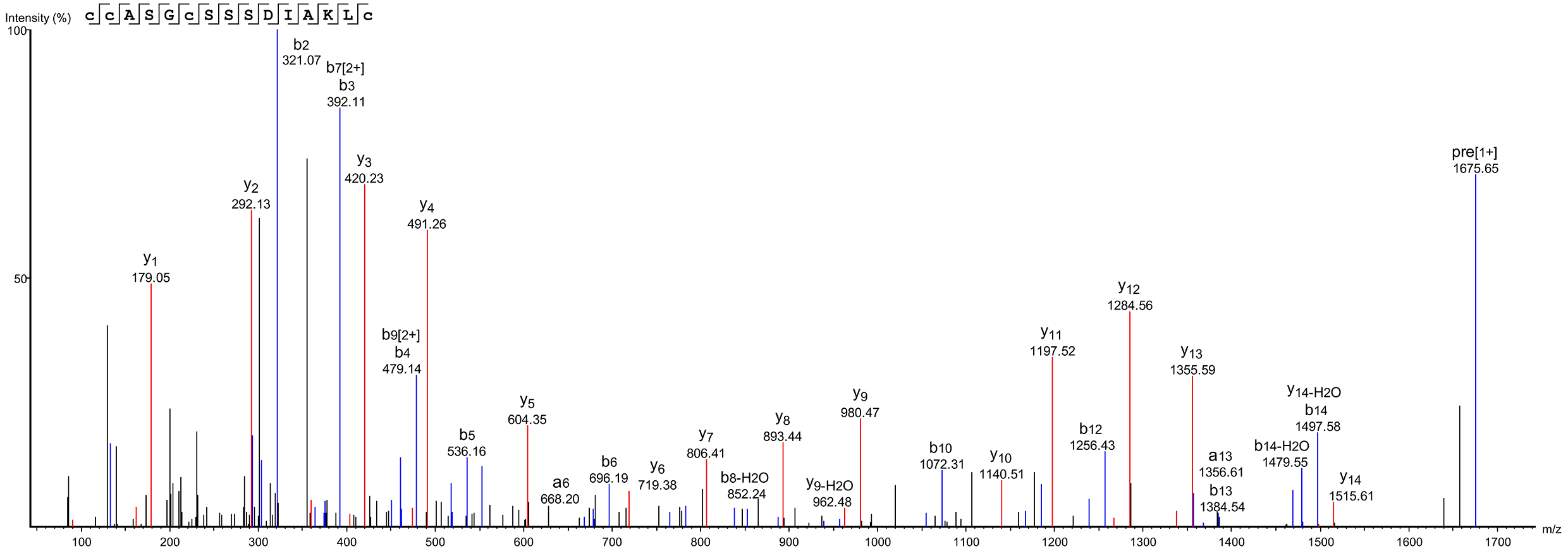


**Peptide CCASGCSSSDIAK: m/z 701.7687**


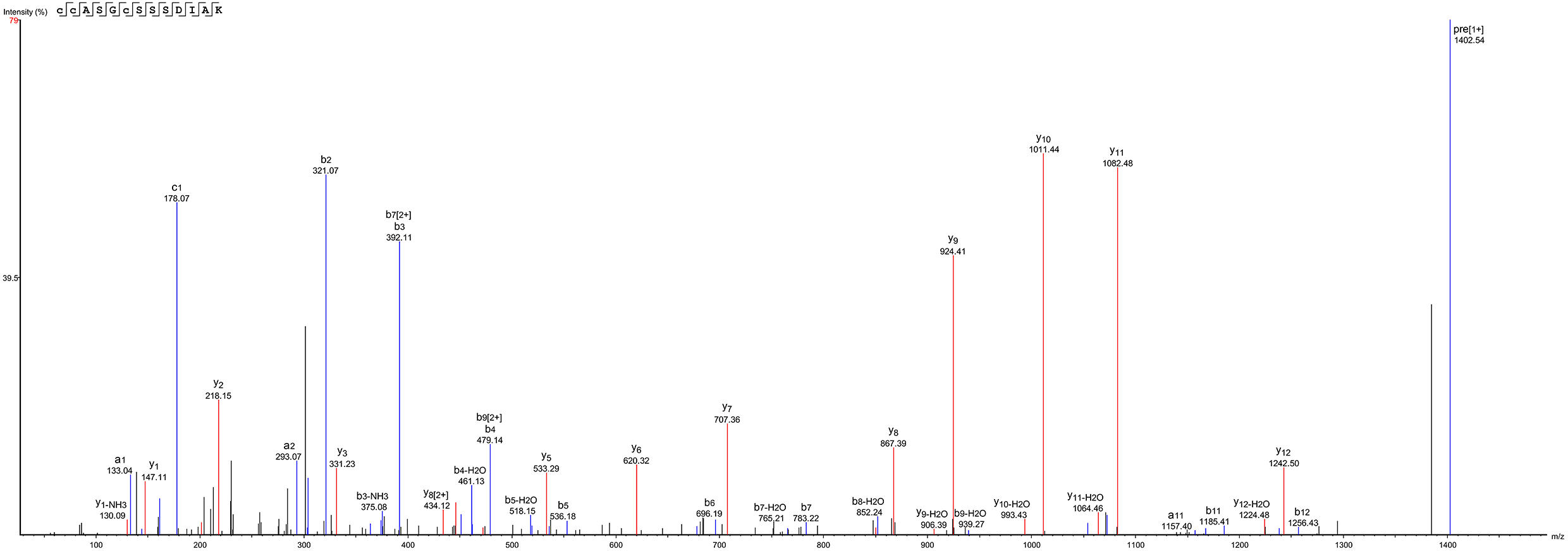


**Peptide LCGADLSR: m/z 446.2192**


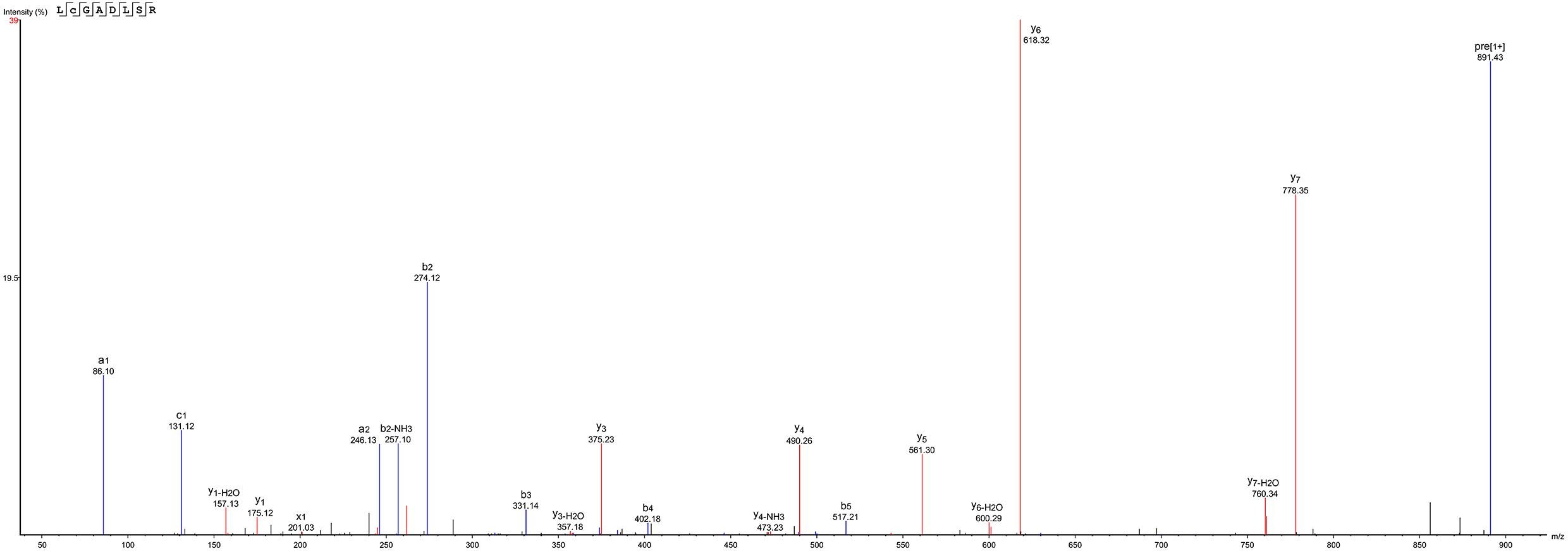


**Peptide ASGCSSSDIAK: m/z 541.7391**


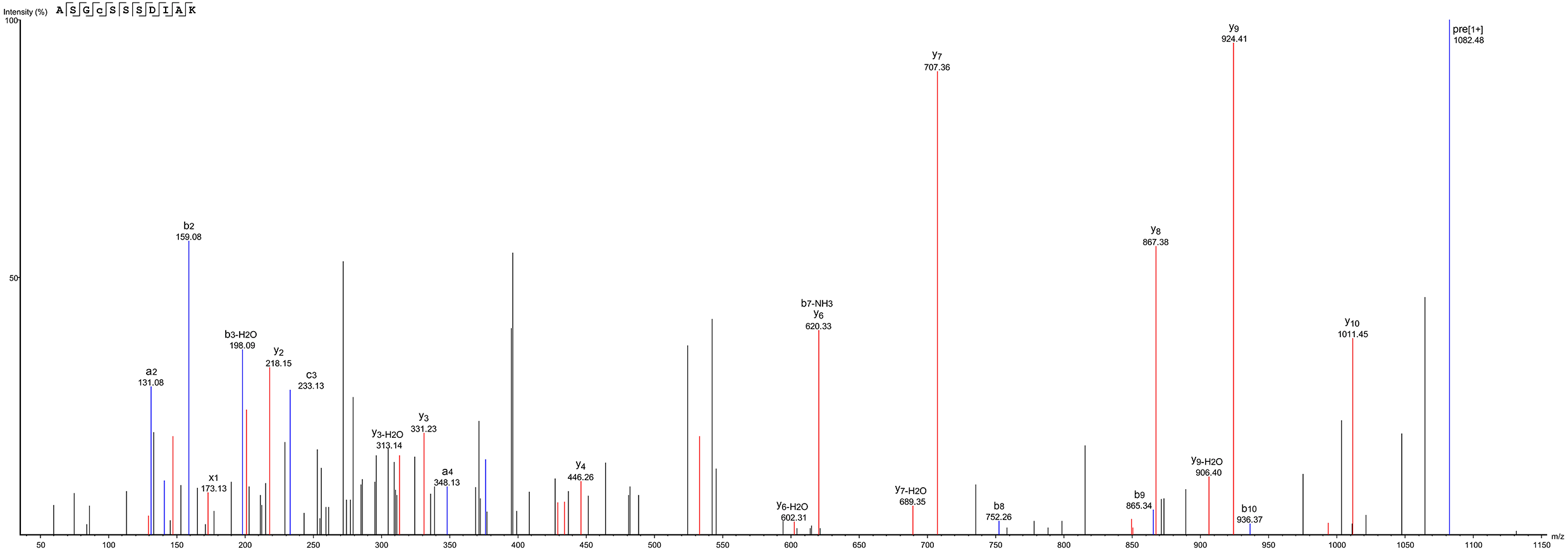


**Peptide CCASGCSSSDIAKL: m/z 758.3106**


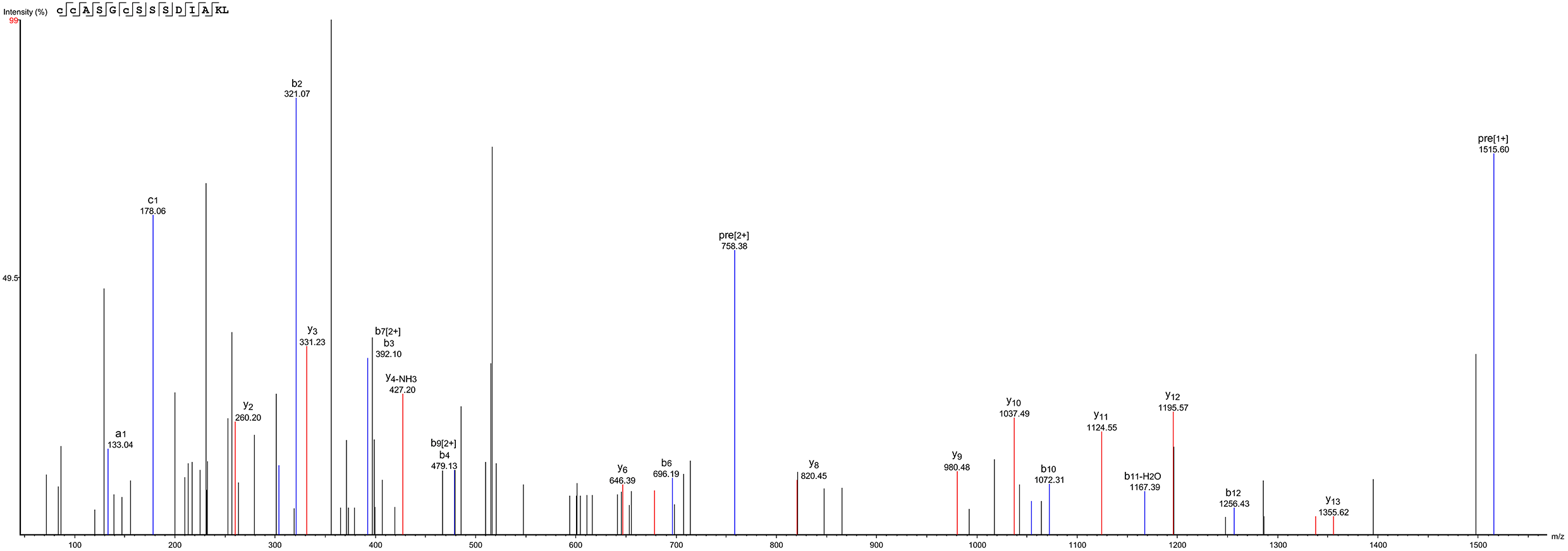


**Peptide GSGSNGGIAR: m/z 438.2333**

**
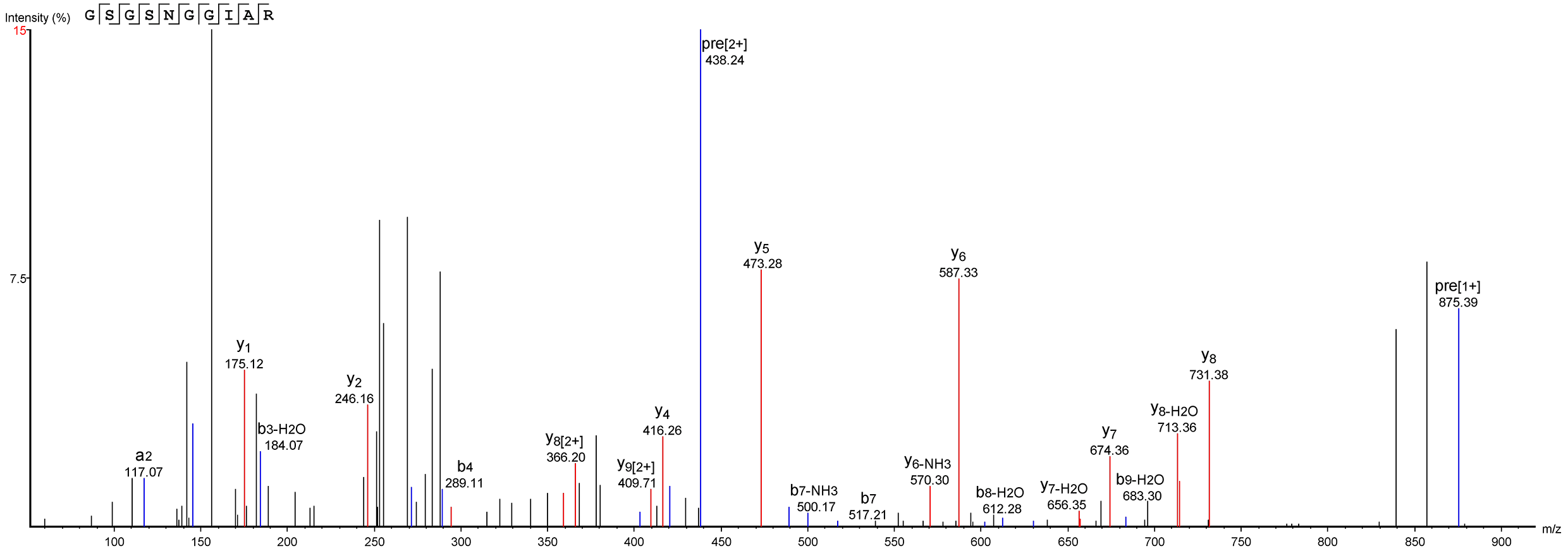
**

**Peptide CCASGCSSSDIA: m/z 637.7233**

**
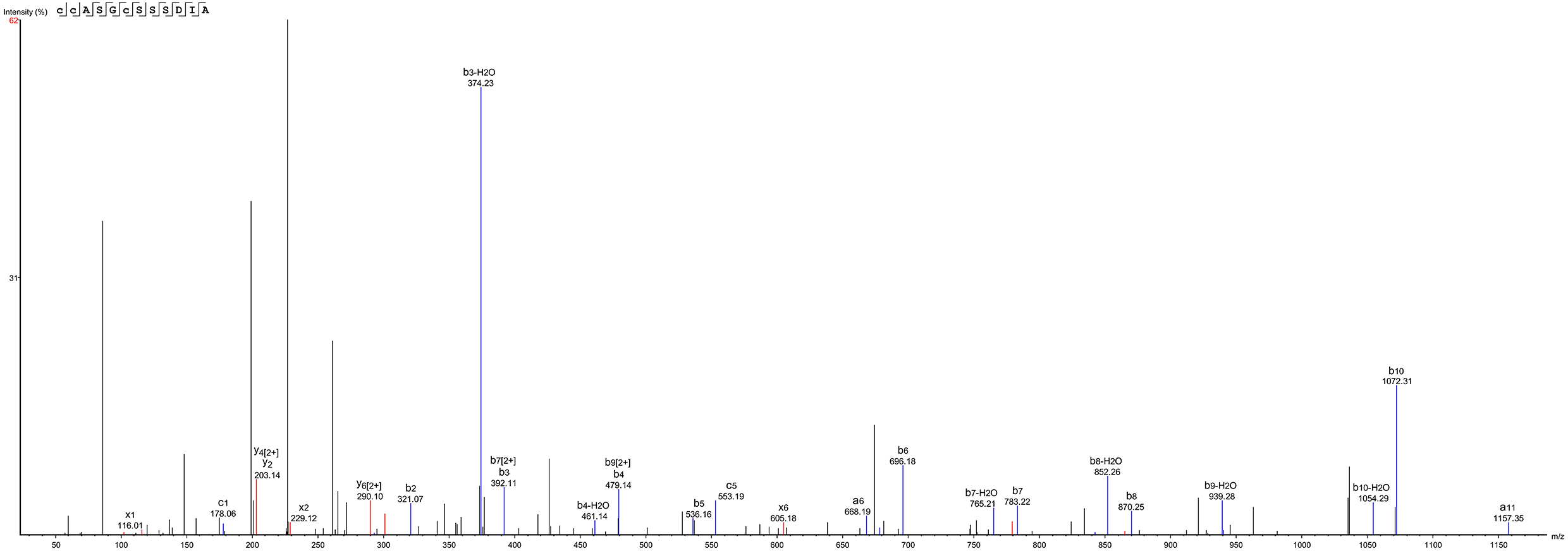
**

**Peptide AVYRVCSH: m/z 496.2416**

**
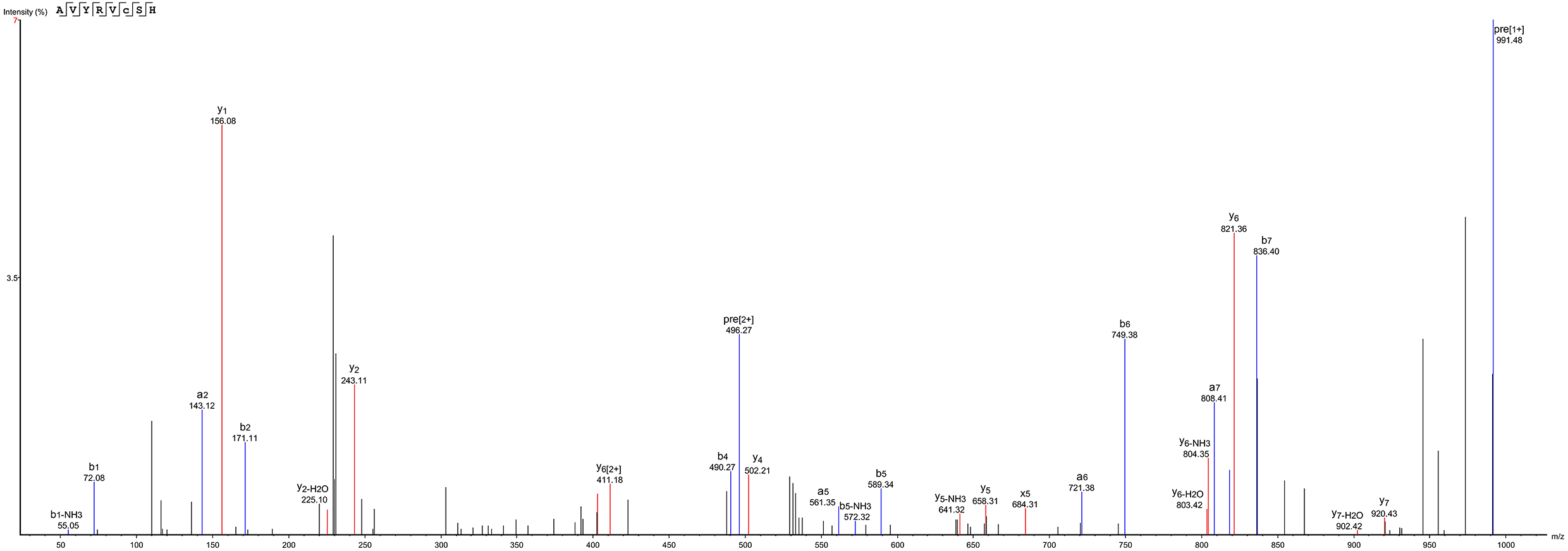
**

**Peptide ASGCSSSDIAKLC: m/z 678.2972**

**
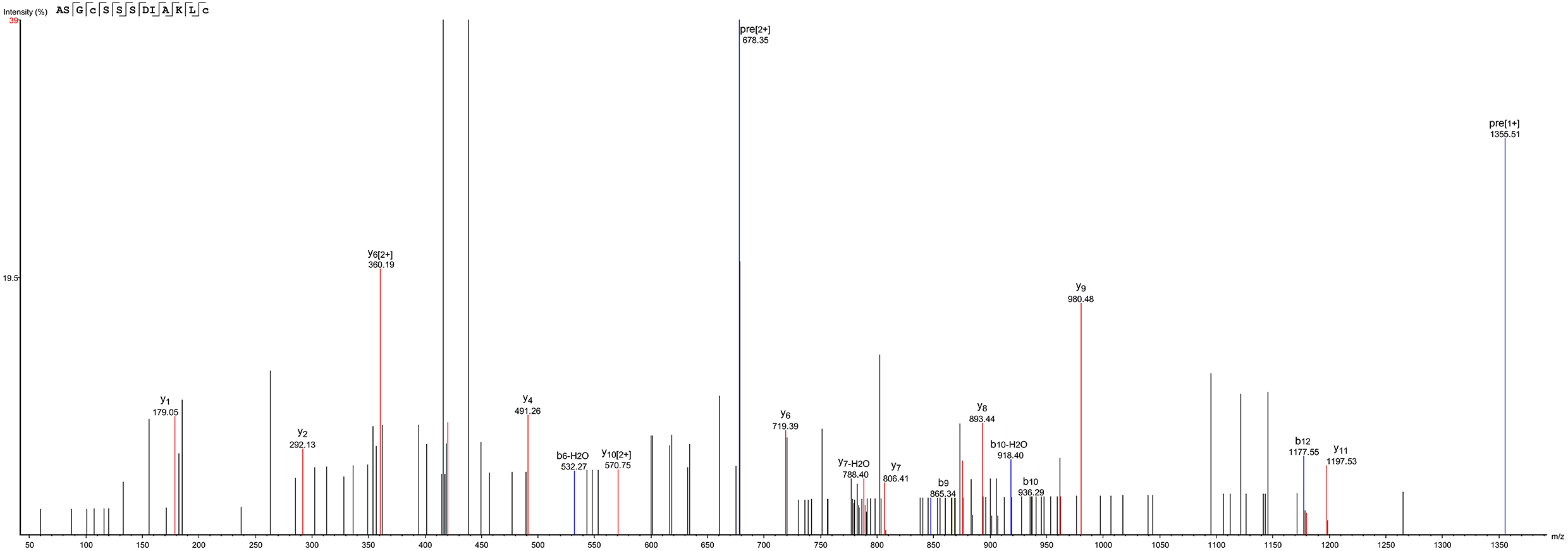
**

**Peptide RLCGADLSR: m/z 524.2709**

**
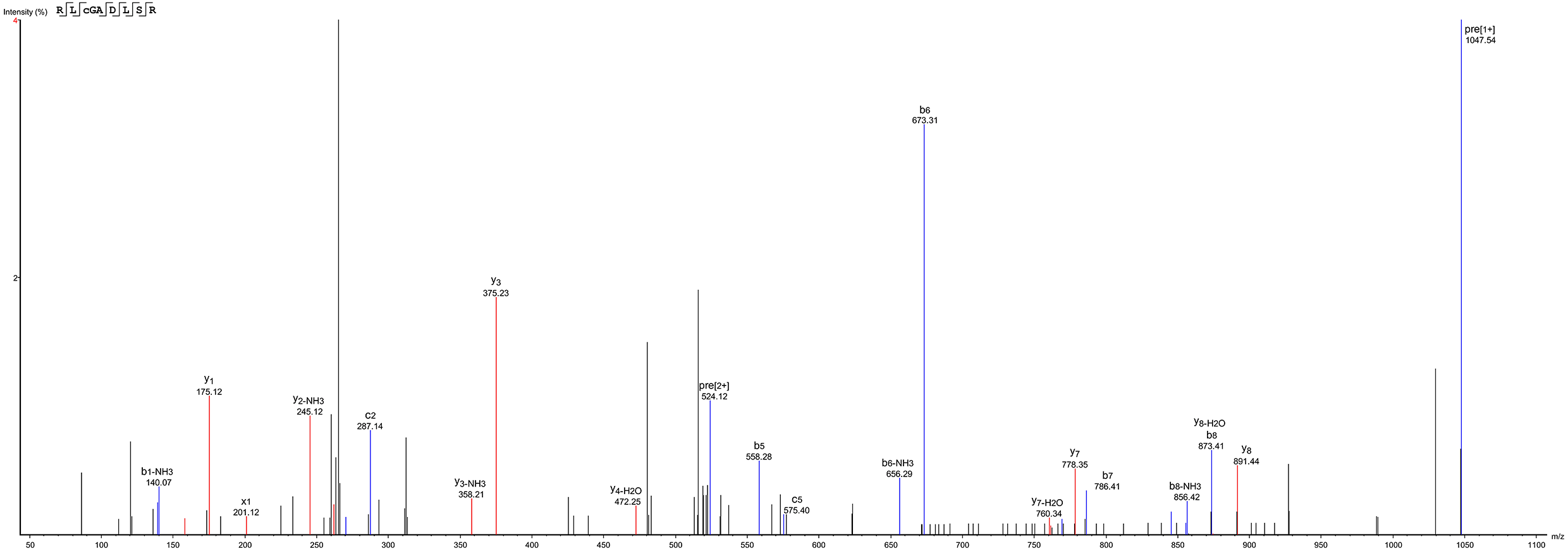
**

**Peptide AVYRVC: m/z 384.1959**

**
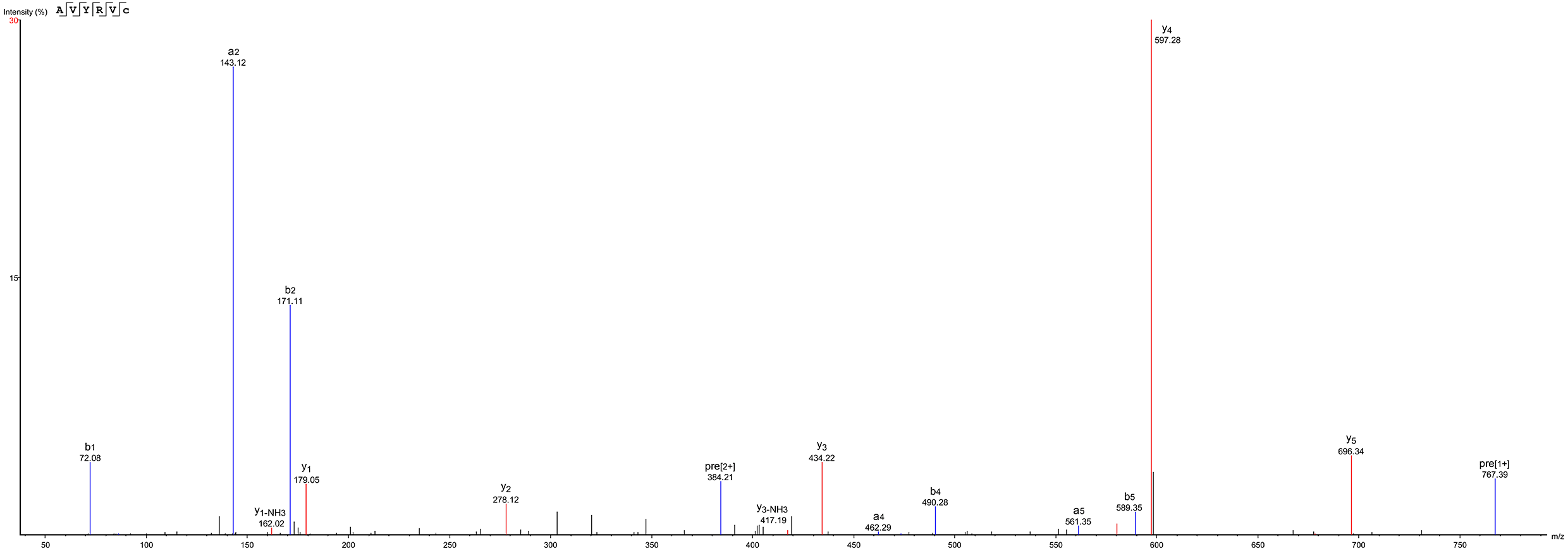
**
