# Supplementary material for: Aquaculture Breeding Enhancement: Maturation and Spawning in Sea Cucumbers Using a Recombinant Relaxin-Like Gonad-Stimulating Peptide
Source: Front Genet. 2019 Feb 19;10:77. doi: 10.3389/fgene.2019.00077 (PMC6389678; doi:10.3389/fgene.2019.00077)
Supplement: Supplementary Figure S1 — Antibiotic screening for high expression of RGP in yeast colonies. [file Image_1.pdf]

frontiers

Supplementary Figure S1: The yeast expression by antibiotic screening of RGP colonies

Article Title:

"Aquaculture breeding enhancement: Maturation and spawning in sea cucumbers using a recombinant relaxin-like gonad-stimulating peptide"

Authors:

Hoang Dinh Chieu[1,2], Luke Turner[3], Meaghan K. Smith[1], Tianfang Wang[1], Josephine Nocillado[1], Peter Palma[1, 4], Saowaros Suwansa-ard[1], Abigail Elizur[1], and Scott F. Cummins[1,*].

Institutional affiliation:

[1] Genecology Research Centre, University of the Sunshine Coast, 90 Sippy Downs Drive, Sippy Downs, Queensland 4556, Australia;
[2] Research Institute for Marine Fisheries (RIMF), 224 LeLai Street, HaiPhong City, Vietnam;
[3] Tasmanian Seafoods Pty. Ltd., Tasmania, Australia;
[4] Aquaculture Department, Southeast Asian Fisheries Development Center, Tigbauan, Iloilo 5021, Philippines.

Address correspondence to:

* Assoc. Prof. Scott F. Cummins. Genecology Research Centre, Faculty of Science, Health, Education and Engineering, University of the Sunshine Coast, Australia. Tel: +61 7 5456 5501; Fax: +61 7 5456 5010; email: scummins@usc.edu.au

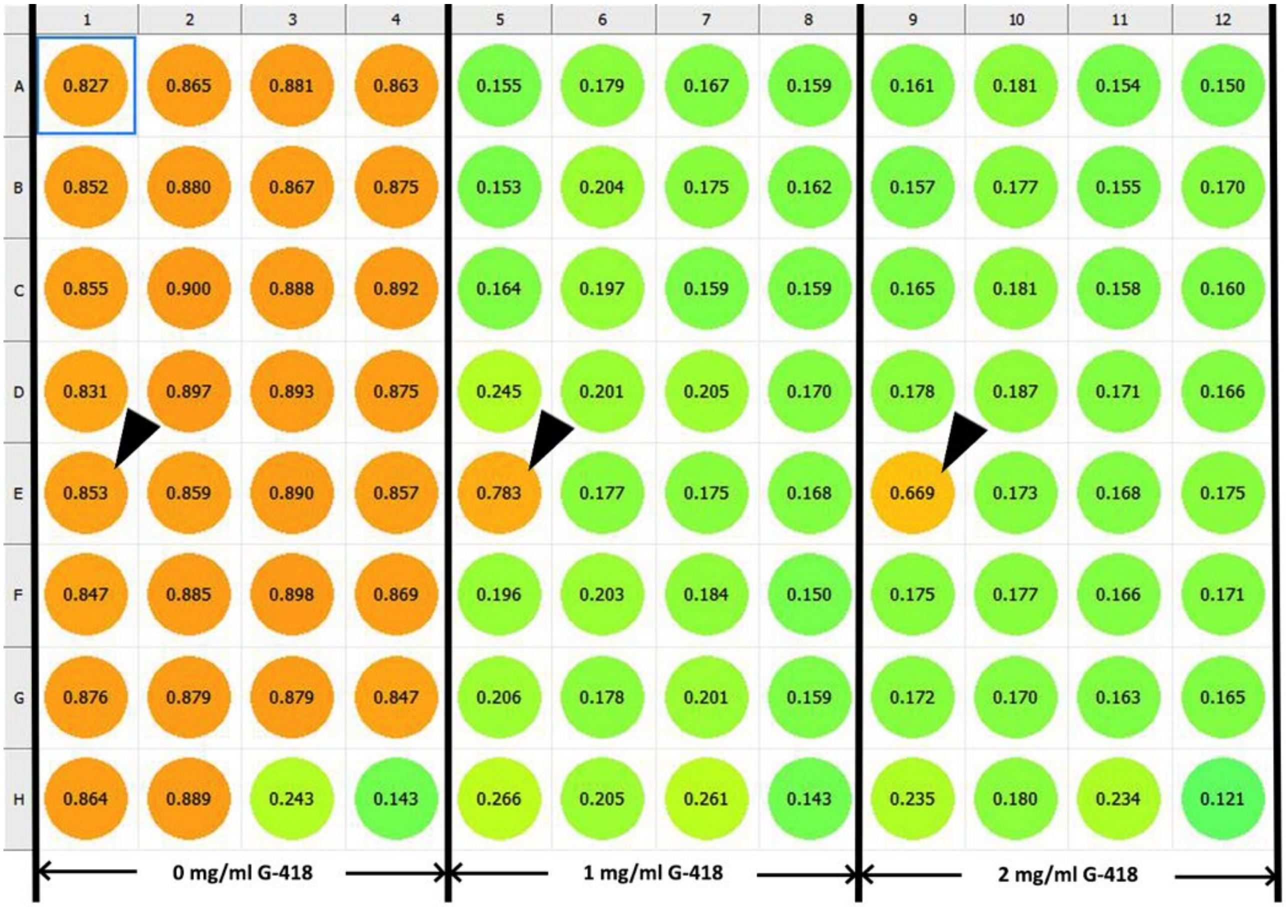

Figure S1. The expression of RGP colonies in a 96-well culture plate based on the highest OD_{600} value in the geneticin concentrations of 1 and 2 mg/mL G-418 antibiotic. Column 1 – 4, 5 – 8, 9 – 12 indicate yeast colonies grown in YPD solution with 0 mg/mL, 1 mg/mL and 2 mg/mL G418, respectively. Arrowheads indicate the wells (E1, E5, E9) containing recombinant RGP. The wells H3, H4, H7, H8, H11 and H12 indicate control group with only YPD solution.
